# Revisiting Curcumin in Cancer Therapy: Recent Insights into Molecular Mechanisms, Nanoformulations, and Synergistic Combinations

**DOI:** 10.3390/cimb47090716

**Published:** 2025-09-03

**Authors:** Khadija Akter, Kainat Gul, Sohail Mumtaz

**Affiliations:** 1 Department of Plasma Bio Display, Kwangwoon University, Seoul 01897, Republic of Korea; santaafrin02@gmail.com; 2 Department of Botany, Hazara University, Mansehra 21120, Pakistan; kainat.botanist38@gmail.com; 3 Department of Chemical and Biological Engineering, Gachon University, 1342 Seongnamdaero, Sujeong-gu, Seongnam-si 13120, Republic of Korea

**Keywords:** chemotherapy, curcumin, cell cycle arrest, nano-encapsulation, nanoparticle-based drug delivery systems, combination therapies

## Abstract

Curcumin has been extensively investigated as an anticancer agent, yet its clinical application remains constrained by low bioavailability, incomplete mechanistic understanding, and limited therapeutic optimization. In light of growing resistance to conventional chemotherapies and the demand for safer, multi-targeted agents, this review revisits curcumin with a contemporary lens. We critically evaluate the literature published since 2020, focusing on newly elucidated molecular mechanisms by which curcumin regulates tumor progression, including modulation of oncogenic signaling pathways (Wnt/β-catenin, PI3K/Akt/mTOR, JAK/STAT, and MAPK), induction of ferroptosis, and epigenetic reprogramming. A particular emphasis is placed on recent advances in nanoformulation strategies that enhance curcumin’s pharmacokinetic profile and target-specific delivery. Furthermore, the emerging paradigm of combination therapy is explored, where curcumin acts synergistically with chemotherapeutics and phytochemicals to overcome drug resistance and potentiate anticancer efficacy. This review identifies key knowledge gaps, such as inconsistent clinical translation and the underexplored interplay between nanocurcumin systems and immune modulation, outlining directions for future translational research.

## 1. Introduction

### 1.1. Overview of Curcumin: Sources and Structure

Curcumin, scientifically designated as diferuloylmethane, represents a hydrophobic polyphenolic compound isolated from the rhizomes of the Curcuma genus, classified within the Zingiberaceae family. This particular genus encompasses species such as *Curcuma longa*, *Curcuma amada*, *Curcuma zedoaria*, *Curcuma aromatica*, and *Curcuma raktakanta.* Among these species, *Curcuma longa*, commonly known as turmeric, is the most extensively utilized. Turmeric rhizomes typically contain 3–5% curcuminoids, comprising mainly curcumin (around 75%), along with demethoxycurcumin (10–20%) and bisdemethoxycurcumin (5%) [1,2].

**Figure 1:**
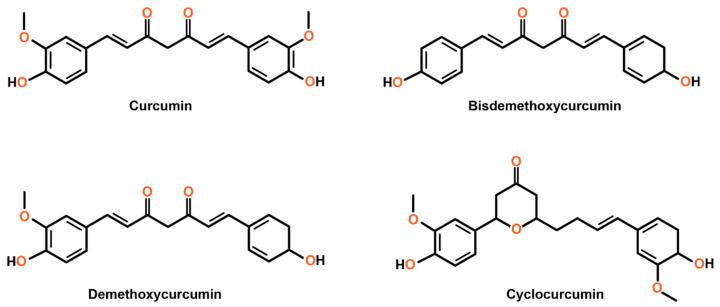
Chemical structure of curcumin and its derivatives. The structure consists of two aromatic rings connected by a chain of carbon atoms with various functional groups. These can be modified to create derivatives with elevated biological and pharmacokinetic properties. The derivatives indicate potential modifications to the curcumin structure, suggesting the versatility of this compound.

Southeast Asian, Middle Eastern, and Indian cuisines frequently use this bright yellow-orange spice. Apart from turmeric, other Curcuma species, like *Curcuma aromatica*, also contain trace levels of curcumin and related curcuminoids. Curcumin, scientifically referred to as diferuloylmethane, possesses the molecular formula C_21_H_20_O_6_, exhibits a molecular weight of 368.385 g/mol, and demonstrates a melting point range between 179 and 182 °C. The compound has a specific gravity of 0.9348 at a temperature of 15 °C and an estimated log Kow value of 3.29. Curcumin typically manifests as bright orange, crystalline particles or granules. Polish chemists first hypothesized the structure of curcumin in 1910 [3]. In the 1980s, researchers identified trace amounts of cyclocurcumin, a fourth natural curcumin analog, in extracts derived from turmeric root (Figure 1) [4].

Curcumin has two phenyl rings containing hydroxyl (-OH) and methoxy (-OCH_3_) groups, which significantly influence its chemical properties and reactivity. These functional groups are particularly important due to their interactions with hydroxyl radicals (•OH), which are highly reactive and have an extremely short lifespan among reactive oxygen species (ROS) in oxygen-respiring organisms [3,5]. Curcumin is classified as a polyphenol, a type of secondary metabolite produced by plants [6]. When exposed to ultraviolet light and certain environmental factors, curcumin quickly breaks down into various compounds, including trans-6-(4′-hydroxy-3′-methoxyphenyl)-2,4-dioxo-5-hexanal, vanillin, ferulic acid, and ferulomethane [3]. However, curcumin remains stable when kept at the recommended temperature of −20 °C [7].

### 1.2. Historical Use in Medicine and Pharmacology

The Curcuma species has a rich historical background rooted in Far Eastern medicine, with its use in Ayurveda spanning approximately 5000 years and in Atharveda for around 2000 years [8,9,10,11]. Turmeric, primarily obtained from *Curcuma longa*, is extensively grown in China, India, and Southeast Asia and has been an integral part of both Traditional Chinese Medicine (TCM) and Indian Ayurveda for centuries [12]. The key phytochemical in turmeric, curcumin, has been found to be helpful in reducing cholesterol for patients suffering from chronic and inflammatory diabetics. It also has cardioprotective effects, possibly due to its ability to reduce C-reactive protein [13,14]. Turmeric, also known as Jiang Huang, has been used as a potent herb in TCM for an extended period. Due to its warm and bitter nature, it is believed to help remove wind from the body, improve circulation and qi flow, and lead to relief from pain and inflammation. Turmeric holds particular significance in TCM, where it addresses ailments linked to blood stasis, including joint pain, menstrual discomfort, and injuries from trauma [15].

In both TCM and Ayurveda, turmeric is recognized as a bitter digestive and carminative. Acting as a cholagogue, turmeric stimulates bile secretion, thus aiding in the digestion of fats. Traditionally, Chinese practitioners have used turmeric to address gallbladder issues, relieve lung congestion, improve blood circulation, facilitate digestion and absorption of nutrients, and normalize menstrual flow [16]. Moreover, curcumin and other natural compounds have long been explored as therapeutic agents due to their diverse bioactive properties, and they are widely investigated for the treatment of cancer and various other diseases, offering potential benefits with fewer side effects compared to conventional therapies [17,18,19,20]. Curcumin, the primary yellow polyphenolic compound in turmeric, exhibits a variety of physiological and medicinal uses, such as neuroprotective, anticancer, antibacterial, antidiabetic, chemoprotective, and immunomodulatory activities [12,21,22,23]. In TCM, turmeric is also used to address jaundice and digestive problems resulting from internal dampness and heat. Its role in “unblocking” meridians and alleviating pain is highly valued, and it is commonly incorporated into remedies for conditions like thoracic pressure and arm discomfort. To enhance its effectiveness and maintain energetic balance, turmeric is often paired with other complementary herbs [24,25,26].

This review explores curcumin’s remedial effects in cancer interventions by analyzing the underlying pathways, clinical evidence, and recent advancements. It highlights strategies to enhance curcumin’s bioavailability, including developing drug transport utilizing nanoparticles and nanoformulations. Additionally, it sheds light on curcumin’s interactions with other bioactive compounds and its role in enhancing chemotherapy effectiveness.

## 2. Curcumin’s Biological Mechanisms

**Figure 2:**
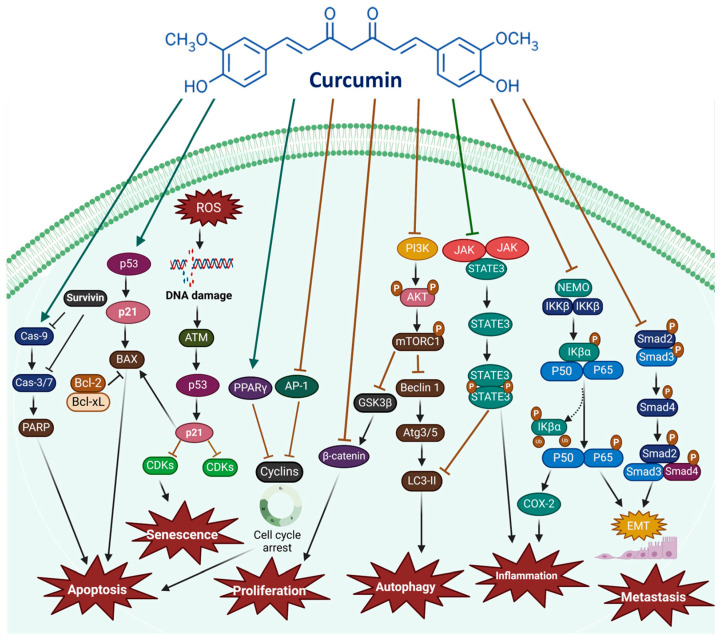
Curcumin plays a pivotal role in cancer by modulating numerous signaling pathways and molecular targets, as indicated by dark teal arrows for upregulation and brown arrows for downregulation. It influences essential mechanisms such as inflammation, ROS production, cell proliferation, apoptosis, autophagy, senescence, migration, invasion, angiogenesis, metastasis, and EMT, thereby shaping cancer progression and therapeutic outcomes [34,35,36]. The figure was prepared using Biorender (https://www.biorender.com/).

Curcumin has attracted considerable interest for its anticancer potential, primarily because it has influenced diverse biological processes implicated in oncogenesis. These pathways regulate fundamental dynamics like cell growth, programmed cell death, angiogenesis, and metastasis—key factors in cancer. By targeting multiple molecular pathways, including Wnt/β-catenin, PI3K/Akt/mTOR, JAK/STAT3, MAPK, NF-κB, and Notch, curcumin suppresses cancer growth and induces apoptosis. Its multifaceted action underscores its broad-spectrum effects, offering a deeper understanding of its capability to combat cancer at a molecular level. As illustrated in Figure 2, curcumin promotes apoptosis by regulating various molecular targets associated with both cellular and receptor-mediated cell death pathways. Therefore, targeting these apoptotic pathways represents an optimistic approach to combating cancer.

### 2.1. The Wnt/β-Catenin Pathway: A Key Driver in Cancer Progression

Wnt/β-catenin signaling, an evolutionarily conserved pathway, plays a critical role in cancer development, particularly in cell proliferation, survival, and stemness [27,28]. Dysregulation of this pathway is linked to tumor initiation, malignant transformation, and poor patient outcomes [29,30]. Dysregulation of this pathway is linked to tumor initiation, malignant transformation, and poor patient outcomes [21,22], as Wnt/β-catenin activity disrupts normal cellular processes and promotes oncogenesis [31,32]; under normal conditions, β-catenin is phosphorylated by GSK3β and CK1α, leading to proteasomal degradation [33]. Binding of Wnt ligands to FZD and LRP5/6 receptors prevents this degradation, enabling β-catenin nuclear translocation and activation of target genes such as Cyclin D1 and c-Myc [33]. Mutations, ligand/receptor dysregulation, or epigenetic changes often contribute to pathway hyperactivation in cancers.

**Figure 3:**
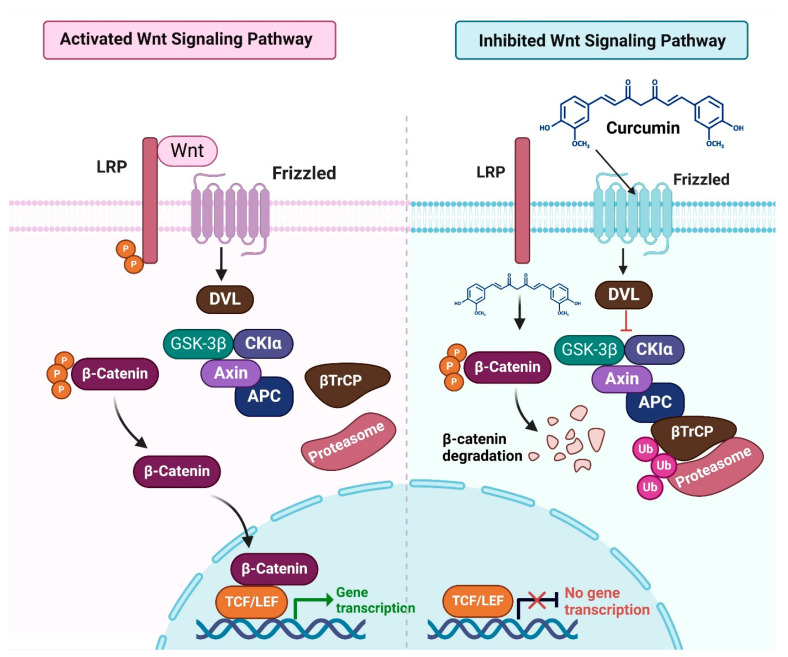
The Wnt/β-catenin signaling pathway is regulated through phosphorylation and degradation processes. When curcumin is introduced, cytoplasmic β-catenin is phosphorylated by a destruction complex composed of APC, AXIN, GSK3β, CK1α, and β-TrCP, an E3 ubiquitin ligase. This phosphorylation by GSK3β and CK1α triggers β-catenin’s ubiquitination and proteasomal degradation. Conversely, when Wnt binds to the FZD receptor, the receptor recruits DVL to the membrane. DVL interacts with AXIN, forming a receptor-bound complex that inhibits the destruction complex’s activity. This inhibition permits β-catenin accumulation in the cytoplasm, followed by nuclear translocation, where it binds to TCF/LEF transcription factors and coactivators to promote the expression of Wnt target genes [45,47]. The figure was prepared using Biorender.

Natural compounds, including curcumin, can modulate Wnt/β-catenin signaling, inhibiting tumor growth and stemness [37,38]. Curcumin reduces Axin2 expression in HCT116 cells and modulates the Wnt/β-catenin pathway, disrupting cell proliferation and survival, thereby contributing to its anticancer effects (Figure 3) [39]. In lung cancer, curcumin inhibits Wnt/β-catenin activity, reduces ROS levels, and induces apoptosis [40]. Chronic inflammation and oxidative stress amplify β-catenin signaling in tumors, but curcumin counteracts these effects by restoring cellular balance [41]. In breast cancer cells, curcumin induces G2/M phase arrest through Wnt/β-catenin modulation, upregulating GSK3β and reducing nuclear β-catenin [42,43].

In ovarian cancer cells, combination treatment with 5-aza-2′-deoxycytidine (DAC) and curcumin significantly reduced nuclear β-catenin levels, disrupting canonical Wnt/β-catenin signaling [44]. This suppression decreased transcription of downstream oncogenic targets, including Cyclin D1 and c-Myc, demonstrating a mechanistic basis rather than a purely descriptive effect. DAC exhibited stronger inhibition of Cyclin D1 and c-Myc than curcumin alone, highlighting β-catenin as a direct molecular target underlying their synergistic action [44]. In HepG2 liver cancer cells, curcumin directly targets β-catenin and key Wnt/β-catenin regulators, including Dvl-2, Dvl-3, and GSK-3β. By decreasing β-catenin levels and modulating GSK-3 phosphorylation, curcumin downregulates downstream oncogenic effectors such as c-Myc and Survivin while upregulating Axin-2, thereby inducing G2/M cell cycle arrest and apoptosis. This establishes a clear mechanistic basis for curcumin’s anticancer activity via targeted disruption of the Wnt/β-catenin signaling pathway [45]. Moreover, β-catenin serves as a direct molecular target of curcumin, whose transcriptional activity is inhibited through ROS-dependent modulation. This inhibition downregulates downstream genes SOX2 and ABCG2, suppresses cancer stem cell properties, and induces apoptosis, providing a clear mechanistic basis for curcumin’s anticancer effects in HCC827 lung cancer spheres [46].

### 2.2. The PI3K/Akt/mTOR Pathway

The PI3K/Akt/mTOR pathway plays a central role in regulating cell proliferation, growth, and survival, and its dysregulation is a hallmark of many cancers [48,49]. Under normal conditions, PTEN blocks PI3K activation by converting PIP3 to PIP2, thereby preventing downstream Akt activation. Loss or downregulation of PTEN leads to hyperactivation of PI3K/Akt signaling, promoting tumor progression. Targeting PI3K alone may cause side effects, so combinatorial strategies, including natural compounds, are of interest [50]. Curcumin has been reported to directly inhibit key molecular targets within this pathway, including PI3K (p110 catalytic subunit), mTORC1, and EGFR, and indirectly modulate the pathway via IKKβ suppression [51,52]. By directly inhibiting PI3K, curcumin reduces PIP3 levels, leading to decreased Akt phosphorylation and reduced phosphorylation of pro-survival proteins such as BAD, thereby promoting apoptosis. Inhibition of mTORC1 by curcumin relieves its suppression of ULK1 and TFEB, activating autophagy in cancer cells [53,54,55,56]. Curcumin-mediated suppression of IKKβ further downregulates mTORC1 activity, reducing proliferative signaling and enhancing apoptotic responses [51,52].

Additionally, curcumin upregulates PTEN expression, restoring its negative regulatory effect on the PI3K/Akt pathway. This results in inhibition of Akt-mediated pro-survival signaling, decreased Bcl-2 activity, and activation of BAX/BAK-mediated apoptosis in gastric cancer and other malignancies [52]. In liver cancer cells exposed to palmitic acid, combined treatment with curcumin and resveratrol suppressed triglyceride accumulation and modulated PI3K/mTOR/STAT3 signaling, while inhibiting HIF-1α and VEGF expression, demonstrating the broader mechanistic impact of curcumin on tumor cell metabolism and survival [57,58]. In lung and kidney cancer cells, curcumin directly downregulates PI3K and Akt activity, leading to mTORC1 inhibition, which in turn induces both apoptosis and autophagy [53,54,56]. In MN9D cells, curcumin regulates autophagic flux by reducing LC3II levels and restoring p62 degradation, preventing excessive autophagy under stress conditions [55]. Overall, curcumin exerts its anticancer effects through direct molecular targeting of PI3K, mTORC1, EGFR, and IKKβ, and through modulation of PTEN, providing a mechanistic basis for apoptosis, autophagy activation, and inhibition of cancer cell proliferation, rather than merely producing descriptive changes in pathway activity [51,52,53,54,55,56,59].

### 2.3. The JAK/STAT Signaling Pathway

Numerous physiological functions, including immunological responses, cell proliferation, apoptosis, and cancer development, are regulated by the JAK/STAT signaling pathway. This intricate pathway is heavily involved in the advancement of diverse pathological conditions, including inflammatory diseases, blood cancers, and solid tumors [60]. The JAK kinase family consists of four distinct non-transmembrane tyrosine kinases: JAK1, JAK2, JAK3, and TYK2 [61].

Curcumin directly targets key components of this pathway, including JAK1, JAK2, and STAT3, inhibiting their phosphorylation and activity. In multiple myeloma cells, curcumin suppresses STAT3 phosphorylation, blocking its nuclear translocation and transcriptional activity. Similarly, in retinoblastoma cells, curcumin downregulates JAK1 and STAT1/STAT3 phosphorylation while modulating miR-99a, which mechanistically leads to apoptotic cell death [62]. In hematological malignancies, dose-dependent inhibition of JAK2 by curcumin reduces STAT3 and STAT5 activity, directly inducing apoptosis [63]. In breast and ovarian cancer models, curcumin’s direct inhibition of STAT3 phosphorylation prevents its nuclear translocation and DNA binding, thereby suppressing transcription of genes related to cell survival, proliferation, metastasis, and chemoresistance [64,65,66,67]. In ovarian cancer, curcumin enhances cisplatin efficacy by inhibiting STAT3-mediated cancer stemness, establishing a mechanistic link between direct molecular targeting of STAT3 and downstream suppression of oncogenic programs [68].

The JAK/STAT signaling pathway serves as a critical mechanism for relaying extracellular signals from cytokines and growth factors, ultimately regulating gene expression and cellular functions such as activation, proliferation, and differentiation [69]. This signaling system is organized around three key components: membrane-bound receptors, JAKs, and STAT proteins [69]. STAT3 phosphorylation is suppressed by curcumin, blocking its nuclear translocation and transcriptional activity in human multiple myeloma cells. By regulating miR-99a expression and reducing the phosphorylation of JAK1, STAT1, and STAT3, curcumin drives apoptotic cell death in retinoblastoma cells [69]. Moreover, curcumin has been widely investigated for its potential therapeutic benefits in breast cancer, especially through its influence on the STAT3 signaling pathway [64,65,66,67]. Curcumin directly inhibits STAT3 phosphorylation, a key step in its activation, in both constitutive and IL-6-inducible conditions. By blocking STAT3 phosphorylation, curcumin prevents its nuclear translocation and DNA binding, thus impairing its role in driving gene transcription related to cancer cell survival and proliferation [64].

However, most of these studies were conducted in vitro, and effective concentrations varied considerably across experimental models. Limited in vivo and clinical data are currently available, highlighting a significant gap in translating these findings to humans. While curcumin shows promising mechanistic effects in preclinical models, its therapeutic potential in cancer and other JAK/STAT-related diseases remains unconfirmed due to the lack of robust clinical trials. Therefore, further well-designed clinical studies are essential to validate curcumin’s efficacy and safety in targeting the JAK/STAT signaling pathway [70,71].

### 2.4. The MAPK Signaling Pathway

MAPK consists of three major signaling cascades: ERK, JNK, and p38 [72,73]. Through varying degrees of influence on the persistence of cells or mortality, the MAPK pathway is essential for the controlling of apoptosis [74]. Furthermore, the MAPK pathway can directly or indirectly influence proteins involved in apoptosis regulation, including caspases and members of the Bcl2 protein family. Curcumin inhibited cell growth and migration via modulation of β-adrenergic receptors in human glioma cell models (LN229 and U87 MG). At an IC50 of 24 μmol/L, curcumin did not exhibit significant cytotoxicity but reduced ERK1/2 activity, resulting in cell cycle arrest and enhanced apoptosis [75]. An altered MAPK pathway plays a crucial role in breast cancer progression and growth. MAP kinases are strongly associated with invasion, metastasis, chemoresistance, and poor prognosis in both triple-negative and hormone-independent HER2+ breast cancers [75,76]. Transforming growth factor-beta 1 (TGF-β1) is strongly linked to cancer invasion and metastasis in advanced-stage breast cancer. Beyond the TGF-β/Smad signaling pathway, TGF-β1 can also promote tumor growth and regulate cell migration and invasion by activating the MAP kinase pathway through Smad-independent mechanisms [77]. In MDA-MB-231 breast cancer cells, non-toxic doses of curcumin (≤10 μM) inhibited the phosphorylation of ERK1/2 and p38MAPK stimulated by TGF-β1 in a concentration- and time-dependent manner [77]. In lung cancer H1299 cell models, the curcumin–piperlongumine hybrid compound successfully inhibited cell growth, induced G2/M phase cell cycle arrest, and triggered apoptosis. These effects were mediated through the regulation of JNK signaling, highlighting its therapeutic potential as a lung cancer treatment [78].

Although curcumin has been shown to modulate ERK, JNK, and p38 MAPK cascades and inhibit cancer cell proliferation and migration in vitro [75,77,78], the effective concentrations vary significantly across studies and cell types. Most evidence comes from cell culture models, with limited in vivo validation. Differences in dosage, bioavailability, and tumor microenvironment could affect translational outcomes. Therefore, while preclinical data are promising, clinical studies are needed to confirm curcumin’s effects on MAPK signaling in humans.

### 2.5. The p53 Signaling Pathway

The tumor suppressor p53 is a pivotal transcription factor that regulates numerous cellular processes [79]. Extensive research indicates that nonfunctional mutated p53 significantly contributes to tumor initiation and progression, correlating with poor clinical outcomes, reduced survival rates, unfavorable prognosis, and chemotherapy resistance in oncology patients [80,81,82]. Apart from its function as a tumor suppressor, p53 is considered a central focus for molecular cancer therapy since it controls vital functions like DNA damage restoration, senescence, and apoptosis. Known as the “Guardian of the Genome,” p53 is essential for preventing the growth of cells with genetic abnormalities, particularly cancerous ones. A recent study demonstrated that curcumin targets and destabilizes various p53 mutants through the ubiquitination pathway in C-33A cervical cancer cells [83]. Curcumin-loaded emulsion nanoparticles (20 µM) induced cell death in pancreatic cancer PANC-1 cells by activating the p53 pathway [84]. In the nucleus, the transcription-dependent mechanism involves p53 interacting with components of the basal transcriptional machinery to enhance the expression of genes like Bax, Noxa, and Puma [85]. In SKBR-3 and MDA-MB-231 breast cancer cells, treatment with curcumin (20 µM) facilitated caspase-dependent apoptosis through upregulation of p53. This was accompanied by elevated levels of Bax and Bid, along with decreased expression of Bcl-2 and Bcl-xL in the treated cells [86]. Additionally, autophagy is activated by curcumin through the Nrf2/p53 axis, providing protection against DPhP-induced cell death in gastric cancer [87]. Furthermore, the role of curcumin in regulating p53 stability in MDA-MB-231 triple-negative breast cancer (TNBC) cells was assessed, where 20 µM curcumin treatment increased the half-life of the p53 protein. This effect was also observed alongside an elevation in NQO1 levels, which may contribute significantly to p53 stabilization [88].

Curcumin’s activation and stabilization of p53 have been demonstrated in multiple cancer cell lines [84,88]. However, most studies were conducted in vitro or in animal models, and mutant p53 variants respond differently, raising questions about the generalizability of these findings. There is insufficient clinical evidence to support therapeutic p53 modulation by curcumin in patients, highlighting the need for translational studies.

### 2.6. Intrinsic and Extrinsic Pathways

Two important processes that control apoptosis, or programmed cell death, are the intrinsic and extrinsic routes. Both are important in cancer. These pathways help determine whether a cell responds to different biological signals, including oxygen depletion, DNA damage, or oncogenic signaling, by either undergoing apoptosis or surviving. When these pathways are dysregulated in cancer, tumor development, susceptibility to cell death, and unregulated cell survival might result. These signals result in the expulsion of cytochrome c and Smac/DIABLO, as well as changes to the mitochondrial membrane. The activity of Smac/DIABLO can be inhibited by inhibitors of apoptosis [89,90]. Through death receptors found on the surface of different cells, such as Fas receptors, DR4/DR5, tumor necrosis factor receptors (TNF-Rs), and TNF-related apoptosis-inducing ligand receptors (TRAIL-Rs), external signals initiate the extrinsic apoptosis pathway.

**Figure 4:**
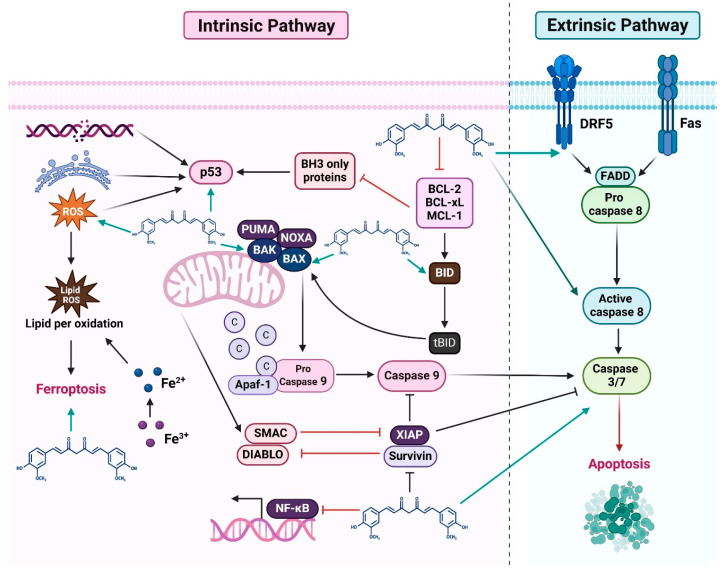
This overview highlights how curcumin modulates intrinsic and extrinsic apoptosis pathways in colorectal cancer cells (CRCs). Curcumin induces apoptosis by activating (blue arrows) or inhibiting (red dashed lines) various molecular targets and signaling pathways. It downregulates antiapoptotic proteins of the Bcl-2 family and apoptosis inhibitors like Survivin and XIAP. Simultaneously, it promotes the production of ROS (ROS) and pro-apoptotic proteins, facilitating mitochondrial cytochrome c (C) release. Additionally, curcumin upregulates Fas and death receptor 5 (DR5), triggering the extrinsic apoptotic pathway. Curcumin also induces ferroptosis, reducing CRC proliferation [95,96,97]. The figure was prepared using Biorender.

Curcumin promotes autophagy in SKOV_3_ ovarian cancer cells by blocking the AKT/mTOR/p70S6K pathway, inhibits their development by activating PARP-1 and caspase-9, and induces apoptosis in a concentration-dependent manner [91]. Curcumin was shown to upregulate the polyamine catabolic enzymes PAO and SSAT, resulting in ROS generation and the activation of both intrinsic and extrinsic apoptotic pathways in breast cancer cell lines MDA-MB-453 and MDA-MB-231, including those expressing growth hormone [92]. Intrinsic apoptosis is initiated by curcumin in acute lymphoblastic leukemia cells, resulting in reduced viability [59]. Specifically, it downregulates AKT and IAPs, increases Bax/Bcl-2 levels, promotes cytochrome c from the mitochondria into the cytoplasm, and activates caspase 3 and PARP-1 cleavage, along with ROS production [59]. The curcumin-induced intrinsic apoptotic pathway was also observed in a neuroblastoma SK-N-SH cell model [93]. In colorectal cancer cells (CRCs), curcumin exhibits anticancer effects by activating intrinsic and extrinsic apoptosis through the modulation of molecular targets and signaling pathways, particularly by preventing the NF-κB pathway. Its actions involve triggering caspase cascades, disrupting mitochondrial membrane potential, and regulating apoptotic proteins. Studies highlight that curcumin upregulates Bax and Bak, downregulates Bcl-2, and enhances cytochrome c, thereby initiating the intrinsic cell death pathway in CRCs [10,94] (Figure 4). Conversely, a key mechanism of mitochondria-independent or extrinsic apoptosis induction by curcumin involves its effect on death receptors. These receptors are essential for transmitting apoptotic signals from the cell membrane to cytoplasmic signaling pathways (Figure 4).

Curcumin induces apoptosis through both intrinsic and extrinsic pathways in diverse cancer models [91,92,93]. However, apoptosis induction is highly dose- and context-dependent. In vitro concentrations are often higher than achievable plasma levels in humans, and protective autophagy mechanisms may reduce efficacy. Clinical validation of curcumin-mediated apoptosis remains limited, emphasizing the need for pharmacokinetic studies and dose optimization.

### 2.7. Curcumin Inhibits Cancer Cell Proliferation and Induces Cell Cycle Arrest

Cell proliferation is the process through which the number of cells increases. In the case of tumors, abnormal proliferation is essential for their growth. A faster multiplication of cancer cells suggests a more aggressive and rapidly spreading disease. Extensive research has shown that curcumin effectively inhibits cancer cell growth and halts cell cycle progression. Studies suggest that curcumin influences numerous pathways and factors linked to tumor cell development in vitro, selectively triggering cell death in cancer cells despite its broad range of targets [98]. Despite its known antitumor properties, the specific molecular roles of curcumin in cancer suppression require further elucidation. According to a study, curcumin effectively reduces colon cancerous cell growth and induces cell death through the p53 pathway [99]. Curcumin causes cell cycle arrest and reduces CyclinA2, CyclinE1, CDK2, and the transcription factor E2F4. Moreover, curcumin inhibited H1975 and PC9 cell proliferation by controlling circRUNX1 [100]. Cyclins are often overexpressed in cancer cells, which results in unchecked cell division. Therefore, a crucial tactic in cancer treatment is to target the cell cycle [101]. A typical characteristic of human malignancies is the dysregulation of cell cycle proteins, contributing to tumor progression [102]. Curcumin promotes apoptosis, autophagy, and cellular senescence in cervical cancer SiHa cells [103]. Similarly, curcumin was found to impede cell growth in ovarian cancer SKOV_3_ cells, causing cell cycle arrest [104]. Similar effects were observed with curcumin in lung cancer H446 and H1299 cells, where it inhibited proliferation and resulted from G2/M phase arrest [78].

Several in vivo studies found that curcumin can suppress tumor growth. In H22 subcutaneous liver tumor models, curcumin effectively reduces tumor size and weight through the suppression of the PI3K/AKT signaling pathway [105]. Curcumin effectively impeded the growth of colorectal cancer SW620 xenograft tumors in nude mice, as evidenced by a decrease in both tumor volume and mass [106]. Another study found that curcumin suppressed ovarian cancer SKOV_3_ proliferation by significantly decreasing the tumor size in nude mice [107].

Curcumin suppresses tumor cell proliferation and induces cell cycle arrest in multiple cancer types [105,106,107]. However, differences in experimental models, treatment duration, and concentration make direct comparisons difficult. Moreover, the majority of studies are in vitro or in xenograft models, with limited clinical trials demonstrating similar effects in patients. Translational studies are required to assess therapeutic relevance.

### 2.8. Apoptosis Induction by Curcumin

Apoptosis is a form of programmed cell death marked by distinct morphological and biochemical changes, such as nuclear fragmentation, mitochondrial membrane permeabilization, cell shrinkage, and the formation of apoptotic bodies [108]. Apoptosis occurs through two primary pathways: the extrinsic pathway, initiated by external signals that activate caspases, and the intrinsic pathway, characterized by mitochondrial membrane permeabilization and cytochrome c (Cyt c) release into the cytoplasm, which subsequently triggers caspase activation and apoptosis [108]. This results in the regulation of ATM/Chk1 kinase, further promoting cell death in BxPC-3 pancreatic cancer cells through post-translational modifications of curcumin, which suppress c-Myc transcription by disrupting the collaborative interaction between Sp1 and hnRNPK, leading to the downregulation of the antiapoptotic protein Bcl-2 and promoting apoptosis [109]. According to different research, curcumin suppressed long non-coding RNA (lncRNA)-LINC00691 levels in B-CPAP thyroid cancer cells. This suppression resulted in higher ATP, lowered glucose uptake, reduced lactate levels, and downregulation of LDHA and HK2 protein expression. When curcumin was combined with siRNA targeting LINC00691, it further elevated ATP levels and inhibited the Warburg effect by suppressing p-Akt activity, thereby promoting tumor apoptosis [110]. Additionally, curcumin treatment resulted in elevated levels of circular RNA (circRNA)-PLEKHM3, which subsequently targeted miR-320a. This interaction led to the downregulation of SMG1, effectively reversing the inhibitory impact of miR-320a on the growth and apoptosis of ovarian cancer SKOV_3_ cells [111].

Mitochondria and ROS are key players in triggering cell death during both healthy and diseased conditions. Enhancing ROS levels can initiate cell death through pathways like necrosis, apoptosis, autophagy, and ferroptosis, which makes increasing ROS a central approach in cancer therapy [112]. Cancer cell death is triggered by curcumin through ROS generation, mitochondrial pathways, and autophagy [113,114,115]. In cervical cancer cells, curcumin induced apoptosis and ROS accumulation. Curcumin treatment elevated cleaved caspase-3 and PARP levels, markers of apoptosis. This effect was attenuated by NAC co-treatment, indicating the critical involvement of ROS in curcumin-induced apoptosis. The administration of Baf-A1, an autophagy inhibitor, reduced autophagy and further enhanced apoptosis, pointing to a protective role of moderate autophagy during stress [103]. Curcumin stabilizes the p53Y220C mutant, enhancing its DNA binding activity. However, the biocompatibility and potential toxicity of nanocarriers require careful evaluation, as certain materials may pose safety concerns in clinical applications [116]. Research demonstrated that curcumin increased ROS in HT-29 and SW480 colorectal cancer cell lines [117].

### 2.9. Ferroptosis Induction by Curcumin

Ferroptosis is a type of non-apoptotic cellular death that is oxidative and dependent on iron. Ferroptosis can be brought on by the depletion of the amino acid cysteine or the suppression of GPX4, in contrast to apoptosis and autophagy, which are the outcomes of certain molecular processes that the cell initiates for its own advantage. This process is connected to the consumption of ATP or the generation of lipid hydroperoxides, which cause catastrophic damage and cell death [118]. Increasing evidence has shown that ferroptosis is essential to the effectiveness of curcumin treatment for a number of malignancies [119,120,121]. Ferroptosis is characterized by the accumulation of intracellular iron, lipid peroxidation, and GSH deprivation [122]. Curcumin regulates ferroptosis by interacting with multiple pathways. Curcumin promotes ferroptosis in colon malignancies by disrupting the PI3K/mTOR pathway. Treatment with curcumin decreases GSH, SLC7A11, and GPX4 levels, while elevating iron, MDA, and ROS, thereby promoting ferroptosis in HCT-8 cells [120]. Another study revealed that curcumin promoted ferroptosis in breast cancer MDA-MB-453 and MCF-7 cells through SLC1A5, which led to increased lipid ROS, higher MDA levels from lipid peroxidation, and elevated Fe^2+^ concentrations inside cells [123]. Mitochondria are the primary sources of ROS within cells and are essential for regulating iron balance and stability [124]. Cells undergoing ferroptosis exhibit distinct morphological changes, such as a reduction in mitochondrial volume and an increase in the density of the mitochondrial membrane [125]. In A549 and H1299 lung cancer cells, curcumin caused mitochondrial membrane rupture, reduced mitochondrial cristae, increased autolysosomes, and elevated Beclin1 and LC3 levels while decreasing P62. Autophagy and ferroptosis induced by curcumin were inhibited by the autophagy inhibitor chloroquine or siBeclin1. In colorectal cancer SW-480 cells, curcumin increased lipid peroxidation levels and ROS accumulation and caused ferroptosis [126].

### 2.10. Curcumin Inhibits Angiogenesis, Invasion, and Metastasis in Cancer Cells

Tumor growth, invasion, and metastasis are all significantly impacted by angiogenesis, the complicated and dynamic process of generating new blood vessels that are regulated by a variety of pro- and anti-angiogenic chemicals [127]. Various genetic products produced by different cells contribute to the process of angiogenesis. Hypoxia is a common condition in tumor environments. HIF-1 is now known to be a crucial regulator of these activities, having first been identified for its function in hypoxia-triggered erythropoietin expression [127]. Proliferation, invasion, and migration of TNBC cells are inhibited by curcumin, including Gli1-overexpressing MDA-MB-231 cells. Furthermore, curcumin reduces the Hedgehog signaling pathway, EMT, and stemness in MDA-MB-231 cells [128]. Furthermore, by focusing on the VEGF/Akt signaling pathway, in combination with omacetaxine, curcumin blocks VEGF/Akt signaling, suppressing U937 lymphoma cell motility, invasion, angiogenesis, and proliferation [129]. A study found that curcumin analog suppresses laryngeal carcinoma cells by modulating the NF-κB pathway [130].

**Table 1:** Molecular pathways in cancer targeted by curcumin.

Type	Cell Lines	Concentration	Target Pathway	Main Finding	Ref.
Breast cancer	4T1 (in vivo)	0.2 mL solution	Ubiquitin-proteasome pathway	Inhibits tumor growth Induces mitochondrial impairment Reduces inflammatory factors and ubiquitination Upregulates myogenic and myo-degradation factor Reduces myostatin in the gastrocnemius	[131]
	MCF-7 (in vitro)	7 μM	miR-15a-5p	Inhibits cellular proliferation and migration Downregulates CCNE1, CHEK1, CDK6, and BMI1gene expression Induces apoptotic cell death, p53, and Bax activation Induces DNA fragmentation	[132]
	T47D, MCF7, MDA-MB-415, MDA-MB-231, SK-BR-3, MDA-MB-468, BT-20 (in vitro)	10 or 30 μΜ	Akt/mTOR signaling pathway	Inhibits cell proliferation Induces apoptosis in breast cancer cells using an in vitro model Triggers cell cycle arrest Hinders the expression of protein kinase B (Akt)/mTOR, BCL2, cleaved caspase 3, CDC25, and CDC2 proteins Increases levels of BAX and p21 protein expression	[133]
	4T1 (in vitro and in vivo)	50 μg/mL	-	Inhibits cellular proliferation Enhances the efficiency of cellular uptake by nanocarriers and their antitumor effects Induces tumor necrosis	[134]
Colorectal cancer	HCT-116 (in vitro)	10, 20, 40 μΜ	CDCA3/CDK1 signaling inhibition	Induce apoptosis Inhibits cell tumor growth and invasion Increases miR-134-5p expressions	[135]
	SW620, HT-29 (in vitro)	5, 10, 20, 40, 80 μΜ	ATF6-mediated endoplasmic reticulum stress	Induce apoptosis Inhibits cell proliferation Increases cleaved caspase-3, BAX, and ATF6 protein expression Increases ERS (related proteins Grp78/CHOP) expression Enhances Ca_2_^+^ concentration in the cytoplasm	[136]
	SW620, LoVo (in vitro and in vivo)	10, 20, 40 μΜ	p53 and SLC7A11/glutathione/GPX4 axis signaling activation	Induce ferroptosis Inhibits cell proliferation Inhibits cell growth and migration Enhances lipid peroxide and malondialdehyde Increases p53, GPX4, SLC7A11 mRNA, and protein expression	[106]
	LoVo (in vitro)	40, 80, 122 μΜ	PI3K/Akt pathway inhibition	Inhibits cell growth and colony formation Inhibits the Akt protein and Bcl-2 mRNA expression Inhibits cell growth and migration Increases caspase-3 and Bax expression	[137]
	HCT-116 (in vitro and in vivo)	10, 20, 30 μΜ	USP4/LAMP3 signaling pathway inhibition	Induces apoptosis Inhibits cell proliferation and invasion Reduces USP4 and LAMP3 protein expression Increases BAX and MMP-2 (matrix metalloproteinase) protein expression	[138]
	HCT-8 (in vitro)	20 μΜ	PI3K/mTOR signaling inhibition	Induces ferroptosis Downregulates GPX4 protein expression Upregulates iron and MDA	[120]
	SW480 (in vitro)	6.29 μM	JAK2/STAT3 pathway inactivation	Induce apoptosis and cell cycle arrest Reduces cell proliferation Increases Bax and Caspase-3/9 mRNA Decreases Jak2 and Stat3 mRNA	[139]
Lung cancer	H446, SBC-2, H1299 (in vitro and in vivo)	6.47 μM	JNK/c-Jun signaling pathway activation	Induces apoptosis and cell cycle arrest Inhibits colony formation	[78]
	A549, H1299 (in vitro and in vivo)	40 µM	ATOX1-mediated copper pathway	Induces apoptosis Inhibits cell proliferation and ATOX1, ATP7A, and COX17 protein expression Enhances copper depletion Inhibits tumor growth and ATP7A and COX17 expression in tumors	[140]
	LK-2, H1650 (in vitro and in vivo)	6.25, 12.5, 25, 50 and 100 μmol/L	DMRT3/SLC7A11 Axis	Inhibits cell proliferation, angiopoiesis, and ferroptosis Downregulates SLC7A11, GPX-4, and DMRT3 protein expression Upregulates TFR1 and ACSL4 protein expression	[141]
	A549 (in vitro)	10, 20, 40, 80 μM	GSH-GPX4 inhibition	Induces ferroptosis Suppresses GPX4 and FSP1 protein expression	[142]
	H1975 and PC9 (in vitro and in vivo)	20, 30, 40 μM	miR-760/RAB3D axis	Induces apoptosis Inhibits cell growth and migration Inhibits MMP2 MMP9, and RAB3D protein levels	[100]
	DOC/A549- and VCR/A549-resistant cells (in vitro)	20, 30, 40 μM	ROS-regulated p38 MAPK Phosphorylation	Induces apoptosis Upregulates p-ERK, p38 MAPK, and eIF-2α protein levels Downregulates p-JNK protein levels	[115]
	A549 (in vitro)	20 μM	EMT signaling pathway	Induces apoptosis, migration, invasion and autophagy Increases E-Cadherin, LC3-II and beclin1 protein levels Downregulates N-Cadherin, Snail, and p62 protein levels	[143]
	A549 (in vitro)	5, 25, 125 nM	Nuclear-cytoplasm translocation of TAZ signaling pathway activation Hippo signaling pathway activation	Reduces sphere formation and sphere size Decreases ALDH level Enhances cisplatin sensitivity and TAZ protein degradation	[134]
Prostate cancer	LNCaP (in vitro)	20, 30, 40 μM	miR-483-3p signaling pathway activation UBE2C signaling pathway inhibition	Suppresses cell growth and migration Inhibits UBE2C protein expression Induces apoptosis Upregulates miR-483-3p mRNA expression	[144]
	PC-4, DU145 (in vitro)	10 μM	m6A-modified circ0030568-FMR1 signaling pathway	Inhibits cell proliferation and migration Reduces FMR1 protein expression Induces apoptosis	[145]
	LNCaP, C4-2, PC3, DU145, C42R (in vitro and in vivo)	4, 8, 12 μM	JARID1D demethylation by regulating EMT and AR signaling pathway	Suppresses cell proliferation and invasion Upregulates JARID1D protein expression Inhibits cancer metastasis in vivo Downregulates N-Cadherin and MMP-2 E-Cadherin expression in vivo Upregulates E-Cadherin expression	[146]
	PC3, LNCaP (in vitro)	10, 15 μM	-	Reduces cell growth Promotes cell death and cell cycle arrest Increases E-cadherin, BAX, and P53 mRNA expression Suppresses SNAIL, VEGFA, and VEGFC mRNA	[147]
	DU145, PC3 (in vitro)	2.5, 5 μg/mL	Akt signaling pathway inhibition	Inhibits cell proliferation and migration Reduces integrin (α3, β1) mRNA expression Increases death receptor (DR) mRNA expression Reduces phosphorylation of Akt protein expression	[148]
Ovarian cancer	SKOV3 (in vitro)	20, 40 μM	NF-κB pathway	Suppresses cell growth Downregulates NF-κB, PRL-3, and IL-6 protein levels Upregulates E-Cadherin protein levels	[148]
	PA1 and A2780 (in vitro)	5, 10 μM	PI3K-AKT pathways	Inhibits cells growth, clonal survival, and cell migration Induces apoptosis and cell cycle arrest Suppresses stemness gene (CD44, OCT4, SOX2, and NANO) expression Upregulates cytochrome c and PARP protein expression Increases ATG5, beclin1 protein expression Upregulates P53, CHK2, and γH2AX protein expression Decreases PI3K and AKT Upregulates phospho-ERK1/2	[149]
	Anglne, HO8910PM (in vitro and in vivo)	4, 6 μM	HCAR1-AMPK-SREBP1 signaling pathway	Inhibits cell growth Induces ferroptosis Downregulates SLC11A2 and GPX4 protein expression Reduces HCAR1/MCT1 protein expression Inhibits tumor growth Induces cell migration	[107]
	SKOV3, A2780 (in vitro)	5 μM	miR-9-5p/BRCA1 signaling pathway	Induces synergistic cytotoxicity Prevents tumor cell growth Promotes cell death and cell cycle arrest Upregulates Bax protein expression Downregulates Bcl-2 protein expression Synergistically suppresses tumor growth in vivo Increases BRCA1 levels	[104]
	SKOV3 (in vitro)	10, 20 μM	NFκB pathway	Induces synergistic cytotoxicity Downregulates Bcl-2 and NFκB expression	[150]
Liver cancer	HepG2 (in vitro and in vivo)	10 μM	VEGF/AKT/PI3K signaling pathway inhibition	Suppresses cell proliferation and migration Reduces VEGF, p-PI3K, and AKT protein levels Increases caspases 3 and caspase 8 protein levels	[105]
	HepG2 (in vitro and in vivo)	2.5, 5, 10 μg/mL	-	Reduces cell growth, angiogenesis, and migration Promotes programmed cell death Upregulates BAX and P53 mRNA expression Downregulates BCL-2 mRNA expression Prevents tumor formation	[151]
	HepG2 (in vitro)	100 μmol/L	-	Induces apoptosis and cell cycle arrest Upregulates Bax and p53 mRNA levels Downregulates Bcl-2 mRNA expression	[152]
Pancreatic cancer	PANC-1 (in vitro)	10, 20, 30, and 40 μM	p53 signaling pathway	Prevents cell proliferation and migration Triggers G2/M arrest Enhances p53, p21, and caspase 3 mRNA levels Decreases Bcl-2 mRNA and protein levels	[84]
	MiaPaCa-2, Panc-1 (in vitro)	5 μM	-	Inhibits cell growth and spheroid formation Promotes cell death and G2/M phase arrest Downregulates Mcl1, Bcl-2, CD44, DCLK1, and cMYC protein expression Upregulates cleaved PARP and BAX protein expression	[153]
	PANC-1, SW1990 (in vitro)	20, 40, and 60 µM	Beclin1 signaling pathway	Inhibits proliferation and colony formation Induces apoptosis and cell cycle arrest Inhibits Beclin1 expression Ubiquitination hypoxia-inducible factor-1α degradation Downregulates KSP70 and KSP90 protein levels Inhibits glycolysis	[154]
	BxPC3, SW1990, and PANC-1 (in vitro)	25, 50, and 100 µM	JNK-mediated Inflammation	Suppresses cell proliferation and migration Inhibits inflammatory mediators IL-1β and COX2 Upregulates JNK protein levels	[155]
Cervical cancer	HeLa, CaSki (in vitro)	20, 40 µM	E6 signaling pathway	Suppresses cell growth and migration Promotes cell death Upregulates E-cadherin, p53, and p21 protein expression Downregulates N-cadherin and Vimentin protein expression	[156]
	SiHa, HeLa (in vitro)	25 µmol/L	ATG3-dependent autophagy	Inhibits cell proliferation and migration Increases LC3 protein expression Upregulates MMP-2, P62, and ATG3 protein levels	[157]
	Hela (in vitro)	15 μM	E6, E7, P53, and Rb pathway	Inhibits cell proliferation Induces apoptosis Reduces E6 and E7 mRNA expression Increases p53 and Rb mRNA expression	[158]

## 3. Preclinical and Clinical Evidence of Curcumin in Cancer Therapy

Curcumin has demonstrated anticancer effects across multiple experimental systems, acting on diverse signaling pathways including STAT3, NF-κB, PI3K/Akt, Wnt/β-catenin, and MAPK, which regulate proliferation, apoptosis, angiogenesis, and metastasis [159,160]. While in vitro studies provide detailed mechanistic insights, their translational relevance is limited, and in vivo and clinical data are essential to confirm therapeutic potential. Preclinical in vivo studies have consistently shown curcumin’s efficacy. In xenograft models of breast cancer, curcumin reduced tumor volume by modulating STAT3 signaling, increasing apoptotic markers (cleaved PARP, caspase-3, -7, -9, Bax, Bid) and decreasing antiapoptotic proteins (Bcl-2, Mcl-1, Bcl-xL) [161,162]. Similarly, in murine models of colorectal cancer, curcumin attenuated inflammatory cytokine production and downregulated COX-2 expression, thereby reducing tumor progression [163]. Additionally, it suppressed colorectal tumorigenesis through the Wnt/β-catenin signaling pathway by downregulating Axin2 [47]. In gastric cancer xenografts, curcumin combined with 5-FU and oxaliplatin significantly inhibited tumor growth through synergistic induction of apoptosis [164]. Moreover, tobacco smoke exposure for 12 weeks activated ERK1/2, JNK, p38, ERK5, MAPK, and AP-1 signaling in mouse stomachs, reduced epithelial markers (E-cadherin, ZO-1), and increased mesenchymal markers (vimentin, N-cadherin). Curcumin treatment (50–100 mg/kg) reversed these molecular alterations, suggesting its potential to prevent gastric cancer associated with tobacco smoke exposure [165]. These preclinical findings highlight curcumin’s capacity to act on multiple molecular targets within the tumor microenvironment, reinforcing the in vitro evidence.

Curcumin has been evaluated in preclinical and clinical studies to determine its safety, bioavailability, and therapeutic potential. It has been evaluated both as a monotherapy and in combination with other drugs in clinical trials. In a phase II trial of 21 patients with advanced pancreatic cancer, daily oral administration of 8 g curcumin for 8 weeks showed clinical activity in two patients, with one achieving stable disease for over 18 months and the other experiencing a temporary 73% tumor regression [166]. In a phase I trial of 14 patients with advanced or metastatic breast cancer, curcumin (up to 6 g/day) combined with standard-dose docetaxel for 7 days was found to be safe and effective [167]. A phase I/II study evaluated oral curcumin, alone or with 10 mg/day bioperine, in 29 multiple myeloma patients at doses of 2–12 g/day for 12 weeks. The combination more effectively modulated biomarkers such as NF-κB (p65), COX-2, and phospho-STAT3, and curcumin showed no significant toxicity [168].

High doses or prolonged exposure may cause hepatobiliary adverse effects by interfering with cholecystokinin signaling. In a dose-escalation study, liposomal curcumin at 120 mg/m^2^ was well tolerated, avoiding changes in red blood cell volume observed at higher doses [169]. Curcuminoids were detected in colonic epithelial tissues of subjects consuming 2.35 g/day for 14 days, confirming absorption and tissue binding [170]. In healthy individuals, curcumin at 3.6 g/day reduced DNA adduct formation in colon biopsies, while doses of 2–4 g/day in smokers decreased aberrant crypt foci only at the higher dose, without affecting Ki67 proliferation index [171,172]. As an adjunct to chemotherapy, curcumin (2 g/day) improved overall survival in advanced colorectal cancer patients receiving FOLFOX, though quality of life and neurotoxicity were unaffected [164]. Another ongoing study is evaluating curcumin versus placebo in combination with FOLFOX in inoperable colorectal cancer [173].

A major limitation in translating curcumin into clinical practice is its poor bioavailability due to rapid metabolism and systemic elimination. To overcome this, novel formulations such as liposomal curcumin, curcumin nanoparticles, and curcumin–phospholipid complexes have been developed, showing improved pharmacokinetics and enhanced anticancer efficacy in both in vitro and preclinical evaluations. Future large-scale, randomized clinical trials incorporating these optimized formulations will be critical to establishing curcumin as a viable adjunct in cancer therapy.

### Anti-Inflammatory and Immunomodulatory Effects of Curcumin

Curcumin’s anti-inflammatory activity plays a crucial role in modulating the tumor microenvironment, which is typically characterized by chronic inflammation and immunosuppression that hinder effective cancer immunotherapy [174]. The tumor microenvironment (TME) is inherently inflammatory, driven by persistent production of pro-inflammatory cytokines (e.g., IL-6, IL-1β, TNF-α), chemokines, ROS, and activation of transcription factors such as NF-κB and STAT3 [175]. This chronic inflammation not only promotes angiogenesis, invasion, and metastasis but also creates an immunosuppressive milieu that facilitates tumor immune evasion [176].

By targeting multiple inflammatory pathways, curcumin effectively reduces pro-inflammatory mediators while enhancing anti-inflammatory signaling. For instance, curcumin inhibits NF-κB and COX-2 activation, decreases secretion of IL-6 and TNF-α, and downregulates inducible nitric oxide synthase (iNOS), thereby suppressing the pro-tumor inflammatory cascade [64,177]. In parallel, curcumin’s antioxidant capacity reduces ROS levels, mitigating oxidative stress that otherwise fuels mutagenesis and malignant progression. Importantly, these anti-inflammatory effects have direct implications for cancer immunotherapy [178]. An inflamed TME often impairs antigen presentation, recruits immunosuppressive cells such as myeloid-derived suppressor cells (MDSCs) and regulatory T cells (Tregs), and induces exhaustion of cytotoxic T lymphocytes (CTLs). By alleviating chronic inflammation and restoring immune balance, curcumin may enhance tumor antigen recognition and T-cell activation, thereby potentiating the efficacy of immunotherapy [179,180].

Emerging evidence also supports curcumin’s role in immunogenic cell death (ICD). A unitized ICD nanoinducer combining curcumin, disulfide-bonded organosilica nanoparticles, and iron oxide effectively induced cancer-cell-specific ICD by depleting glutathione, generating hydroxyl radicals, and disrupting Ca^2+^ and thioredoxin reductase. This synergistic activity elevated oxidative and endoplasmic reticulum stress, triggering systemic antitumor immunity with higher selectivity and potency compared to conventional ICD inducers such as doxorubicin [181]. Furthermore, curcumin has been shown to suppress activation of the NLRP3 inflammasome by modulating NF-κB signaling, thereby reducing IL-1β secretion. In malignant mesothelioma, its anticancer effects have been associated with regulation of inflammasome-related pathways, including IL-1 and NF-κB [182]. Similarly, in human chronic myelogenous leukemia (K562) cells, curcumin treatment downregulated IL-6, TLRs, IL-3, and STAT-1 expression [183].

Clinical studies confirm these findings. In a randomized, double-blind trial involving 80 patients with solid tumors, curcuminoid supplementation (180 mg/day for 8 weeks) significantly lowered circulating inflammatory mediators such as interleukins, TNF-α, and MCP-1 compared with placebo [184]. Additionally, in a phase I clinical trial of the polyphenol-based botanical drug APG-157, which includes curcumin, oral cancer patients exhibited reduced salivary levels of IL-1β, IL-6, and IL-8 within 24 h of treatment [185]. Despite its promising anti-inflammatory and immunomodulatory effects, curcumin’s clinical translation is limited by its poor bioavailability, rapid metabolism, and low systemic stability. Therefore, advanced formulations and delivery strategies are required to fully harness its therapeutic potential.

## 4. The Significance of Curcumin in Cancer Prevention

Free radicals and toxic byproducts generated from oxidative stress are significant contributors to cancer progression. Consequently, antioxidants can be crucial in cancer prevention. Curcumin effectively neutralizes free radicals, thereby helping to inhibit cancer initiation [186]. By neutralizing these harmful molecules, curcumin may effectively prevent the onset of cancer. Since curcumin regulates various cellular functions involved in cancer development, it is a powerful tool for cancer prevention. Curcumin can lower the risk of cancer development and progression by investigating pathways related to inflammation, oxidative stress, and cell proliferation [187]. NF-κB contributes significantly to oxidative stress and nitric oxide synthase activity, driving cancer progression. When it is activated, an inflammatory reaction may result, creating an atmosphere that is favorable for the growth of tumors [188]. Curcumin prevents cancer initiation, preventing the formation of NF-κB, which is known to be involved in inflammatory responses associated with tumor development [189,190]. Additionally, curcumin has been shown to influence liver enzymes, particularly cytochrome P450, which is involved in removing toxins [191]. Through these dual actions, curcumin effectively prevents tumor formation and growth by regulating both phase I and II enzymatic pathways [192].

Recent progress in detecting cancer and increasing treatment alternatives has resulted in lower cancer death rates. The increase in drug-resistant cancers emphasizes the pressing demand to investigate and design new and more effective treatments [193]. In this context, curcumin emerges as a promising candidate for an effective anticancer agent, whether used alone or in conjunction with other drugs. It influences various signaling pathways and molecular targets critical to the progression of multiple malignancies (Table 1).

## 5. Combination Therapy: Synergistic Effects of Curcumin with Chemotherapy and Nanoparticle-Based Drug Delivery Systems

**Figure 5:**
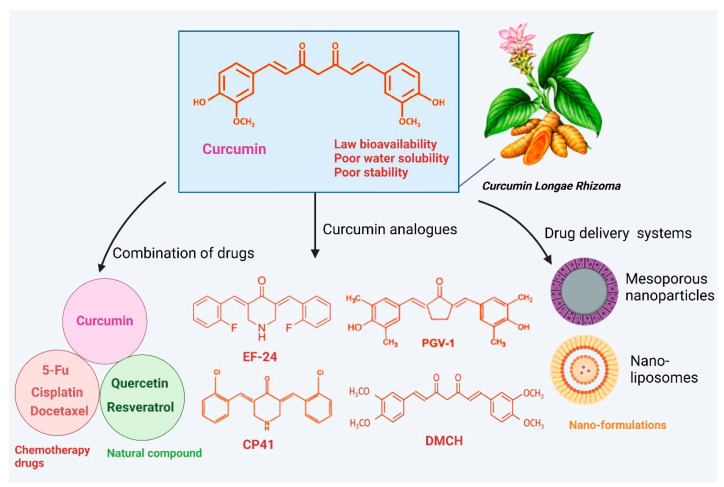
The use of curcumin in cancer treatment is limited by its poor bioavailability. However, this challenge can be addressed through various strategies, including the development of curcumin analogs with improved bioavailability, nanoparticle-based drug delivery systems, combination therapies with other agents, and similar innovative approaches [198,216,217]. The figure was prepared using Biorender.

Although curcumin exhibits promising anticancer activity in studies, its therapeutic use is constrained due to low solubility, stability, and absorption, which can be improved using methods such as advanced analogs, nanoparticle systems, or combination treatments (Figure 5). Combination therapy offers a promising approach to overcome drug resistance, reduce severe adverse outcomes, and enhance the efficacy of cancer treatments. In this context, extensive research has explored the potential of phytochemicals as adjuvants to conventional chemotherapy, yielding promising outcomes. Curcumin is one such compound known for its apoptotic properties and its ability to inhibit multi-drug resistance mechanisms in cancer cells, thereby amplifying the anticancer effects of chemotherapy drugs and reducing the risk of resistance. Chemotherapy drugs such as cisplatin, docetaxel, irinotecan, 5-fluorouracil (5-FU), and paclitaxel (PTX) are widely used for the management of several cancer forms. Their effectiveness is frequently limited by the development of drug resistance, lack of selectivity, and the presence of significant adverse effects. Cisplatin is among the most commonly utilized chemotherapy agents for treating various types of cancer. Curcumin, in combination with cisplatin, suppresses colon cancer. The findings revealed that curcumin significantly enhanced cisplatin’s ability to prevent colon cancer (combination index < 1) compared to either curcumin or cisplatin used individually. Additionally, cisplatin-resistant colorectal cancer cells exhibited a marked increase in glutamine metabolism, indicating a glutamine-addicted phenotype [194]. Curcumin counteracts cisplatin resistance in ovarian malignant cells by regulating PI3K signaling. An in vivo study demonstrated that administering curcumin followed by cisplatin led to the complete regression of tumor mass in induced breast cancer. Furthermore, curcumin positively influenced the expression of Par4, a tumor suppressor gene whose reduced expression is linked to poor prognosis [195].

Additionally, the co-administration of curcumin and DTX demonstrated enhanced cancer-fighting effects by boosting the immune response in head and neck cancer [196]. The combined effect of curcumin and docetaxel on the PI3K/AKT/mTOR pathway induces autophagy and apoptosis in esophageal squamous cell carcinoma [197]. Chemotherapy resistance is a leading cause of death in colorectal cancer. Curcumin promotes chemosensitivity of colon cancer cells to 5-FU [198]. Through the regulation of the PI3K/AKT/mTOR pathway via MACC1, curcumin suppressed colorectal cancer cells, decreasing their resistance to 5-Fu [199]. The potential of curcumin to enhance the anticancer effects of 5-FU was also studied in hepatocellular carcinoma cells. Curcumin significantly enhances the cytotoxic effects of 5-FU on HCC cell lines through synergistic actions, mediated by the suppression of the PI3K/AKT/mTOR pathway in vitro. Moreover, curcumin improves the anticancer activity of paclitaxel in ovarian cancer [104].

It is also worth noting that natural compounds like resveratrol, when used alongside chemotherapeutic drugs, help decrease drug resistance and mitigate the adverse outcomes associated with chemotherapy [200,201]. The impairing of curcumin and resveratrol notably enhances the sensitivity of ovarian cancer, reduces doxorubicin-induced cardiotoxicity, and offers a promising approach for prostate cancer [202]. Curcumin and resveratrol work synergistically to prevent cell death through induction of ER stress, enhanced ROS production, and modulation of autophagy [203]. Furthermore, curcumin and quercetin were more effective in inhibiting cancer growth and inflammation than when used individually in prostate cancer treatment. Their combination significantly reduced proliferation, arrested the cell cycle, and induced apoptosis, demonstrating stronger synergistic effects than either drug alone. Additionally, through their antioxidant properties, the curcumin and quercetin combination modulated various inflammation-related signaling pathways (such as ROS, nitric oxide, and pro-inflammatory cytokines), thereby protecting cells from molecular changes that could trigger carcinogenesis [148]. Additionally, curcumin and quercetin synergistically induce apoptosis in K562 leukemia cells [204].

Furthermore, nanodrug carriers that co-deliver curcumin and chemotherapeutic agents to targeted sites further enhance the effectiveness of treatment. DTX is a commonly utilized chemotherapy drug for the treatment of multiple types of cancer. Nanocarriers, which can encapsulate lipophilic drugs, provide an effective strategy to enhance solubility, minimizing drug interaction with the gut lining. Mixed micelles loaded with DTX and curcumin enhanced drug absorption. They greatly improved bioavailability and cytotoxicity in the treatment of cancer [205]. Another study found that nanodelivery of 5-fluorouracil and curcumin via RGD-decorated nanoliposomes enables synergistic chemotherapy for breast cancer [206]. Curcumin nanoformulations and their associated drug delivery systems are provided in Table 2.

Growing evidence indicates that certain synthesized curcumin analogs have improved solubility and bioavailability, offering greater efficacy against cancers compared to curcumin itself. For instance, CP41, a new curcumin analog, triggers cell death in endometrial cancer through increasing cellular stress [207]. Additionally, the safety assessment of CP41 revealed that it caused negligible adverse effects in mice [207]. Curcumin analog DMCH significantly promotes cell death in HT29 cells [208]. The curcumin analog EF-24 triggers autophagy, leading to ROS-mediated mortality in breast cancer [208]. In liver cancer cells, the curcumin derivative PGV-1 promotes cell cycle arrest [209]. Additionally, curcumin analogs such as WZ35 and CH-5 demonstrate significant inhibitory effects on gastric cancer and osteosarcoma, respectively, through various molecular mechanisms [210,211].

## 6. Curcumin with Combined Treatments in Clinical Trials in Cancer

Ongoing and completed clinical trials have explored curcumin’s potential both as a monotherapy and in combination treatment strategies. Despite its low bioavailability and inconsistent pharmacokinetic and pharmacodynamic data, curcumin has undergone evaluation in initial clinical studies for colorectal, breast, pancreatic, and hematological malignancies. One clinical trial focused on assessing the safety and tolerability of combining immunotherapy with bevacizumab (the active ingredient in Avastin) and chemotherapy with FOLFIRI (folinic acid, 5-FU, and irinotecan) alongside curcumin. Patients in this study received a daily oral curcumin supplement in the form of a ginsenoside-modified nanostructured lipid carrier (G-NLC), which was designed to enhance solubility and oral bioavailability [212,213]. The study monitored extended survival and side effects, showing that curcumin combined with FOLFIRI was well tolerated and did not cause significant harm. Although long-term survival rates were control comparable to the control group, curcumin improved chemotherapy compliance [214]. Another phase I study in patients with advanced colorectal cancer investigated the impact of curcumin on dose-limiting toxicity and the pharmacokinetics of irinotecan, a drug used to treat various solid tumors. Irinotecan undergoes complex metabolism, converting into the active metabolite SN-38, which inhibits topoisomerase II and blocks DNA replication [215].

The pharmacokinetics of irinotecan with higher doses of curcumin were assessed using parameters such as AUC (area under the curve), Cmax (maximum concentration), and Tmax (time to maximum concentration). The findings indicated that curcumin did not affect irinotecan’s pharmacokinetics (NCT01859858) [218]. The combination of oxaliplatin and curcumin was found to significantly reduce cell proliferation and induce apoptosis. In an early-stage clinical study, curcumin doses were gradually increased from 500 mg to 1 g, and finally to 2 g, with no adverse effects observed when combined with chemotherapy. About 80% of patients experienced no adverse effects [219]. A subsequent phase II trial evaluated the effects of curcumin on side effects caused by FOLFOX chemotherapy, along with improvements in disease response and survival. This trial concluded that combining curcumin with FOLFOX chemotherapy is safe and tolerable, with potential benefits for cancer patients (NCT01490996) [164,173]. Currently, several clinical trials are evaluating curcumin’s use in breast cancer treatment. One study investigated the effects of intravenous curcumin alongside chemotherapy for breast cancer. Adding curcumin to paclitaxel significantly increased the response rate to 51%, compared to 33% with a placebo [220]. In pancreatic cancer treatment, the combination of gemcitabine and curcumin (2000 mg/day) proved to be more therapeutically effective than gemcitabine alone [221]. Comprehensive information on several clinical trials with curcumin is available in the previous article [222].

### Bioavailability and Delivery Challenges

Bioavailability refers to the proportion (percentage) of an administered drug dose that successfully enters the circulatory system and becomes available in systemic circulation [223]. For any medicinal product to be effective, the active ingredient must effectively enter the body. Curcumin has poor water dispersibility and a strong affinity for lipids, meaning it dissolves more easily in fats than in water. Its solubility is especially limited in acidic or neutral pH environments, while it becomes more soluble in alkaline conditions. This characteristic challenges curcumin’s bioavailability in the body, as it limits absorption in aqueous environments like the gastrointestinal tract [224]. Curcumin degrades quickly in basic conditions, having a very short life span, and also undergoes photodegradation in organic solvents. When taken orally, approximately 80% of curcumin remains unchanged as it passes through the digestive system. Of the fraction that is absorbed, the majority undergoes a swift biochemical transformation in the gut lining and liver [224]. Clinical studies indicate that even when taken orally at high doses (8 g/day), curcumin is rapidly converted to metabolites, leading to minimal free curcumin levels in plasma (<2.5 ng/mL). This rapid metabolism and low systemic presence limit its therapeutic potential, necessitating strategies to enhance its absorption and bioavailability for effective use in clinical settings [225].

Several promising strategies are being explored to enhance curcumin’s bioavailability, with one key approach involving its integration into nano-sized delivery systems. This method is under investigation in early stage studies, as nanoparticle encapsulation may improve curcumin absorption, stability, and sustained release, ultimately increasing its therapeutic efficacy [226,227,228]. Nano-encapsulation significantly improves curcumin’s dissolution, chemical resilience, and absorption efficiency [229]. Studies show that nano-encapsulated curcumin retains its anti-inflammatory effects more effectively than unformulated curcumin [230,231]. Additionally, nano-encapsulation reduces curcumin’s systemic clearance, extending its half-life in the bloodstream and decreasing its susceptibility to chemical degradation, thereby increasing therapeutic efficacy [230,232].

Additionally, scaling up from laboratory synthesis to large-scale production presents challenges in reproducibility, cost-effectiveness, and consistent therapeutic outcomes, given patient-specific factors such as metabolism, disease stage, and genetic variability [233]. Variations in curcumin concentration across different nanoparticle formulations and the absence of appropriate controls, such as drug-free or functionalized nanoparticles, can introduce bias in efficacy evaluation and complicate interpretation of results. Preclinical studies provide encouraging evidence of efficacy. For example, curcumin-loaded mesoporous silica nanoparticles functionalized with polyethyleneimine–folic acid showed superior tumor inhibition compared to free curcumin in MDA-MB-231 xenograft models, with no apparent toxicity [234]. Similarly, curcumin-loaded magnetic nanoparticles enhanced tumor inhibition in HPAF-II pancreatic cancer xenografts and improved in vivo tumor targeting, as confirmed by immunohistochemistry and imaging studies [235]. Despite these promising preclinical results, few studies have systematically assessed curcumin pharmacokinetics, long-term toxicity, or translational relevance in humans. Differences between in vitro and in vivo performance, along with the limited number of clinical studies, underscore the need for rigorous clinical trials to validate the safety, efficacy, and optimal dosing of curcumin-based nanocarriers [236].

## 7. Enhancing Curcumin’s Efficacy Through Formulation and Nanotechnology in Cancer

Curcumin has demonstrated promising efficacy in preclinical studies by modulating multiple signaling pathways involved in proliferation, apoptosis, angiogenesis, and metastasis [159,160]. Several clinical trials also suggest its potential benefit in various cancers, although outcomes are often limited by poor absorption and rapid systemic clearance [166,167,168]. In terms of toxicity, curcumin is generally regarded as safe, even at relatively high oral doses (up to 8–12 g/day in clinical trials), with only mild adverse effects such as gastrointestinal discomfort being reported [20]. In a mouse model of familial adenomatous polyposis (ApcMin/+) [237], dietary curcumin (0.2–0.5%, 300 mg/kg) significantly reduced intestinal tumor burden by 40%, particularly affecting small- and medium-sized adenomas, while the lowest dose (0.1%) was ineffective. These results, later supported by Park et al. [238], underscore curcumin’s efficacy as a chemo-preventive agent in colorectal cancer. Several studies indicate that curcumin has very low systemic bioavailability after oral administration due to poor gastrointestinal absorption. For example, even high oral doses in rats and humans result in only trace or undetectable serum concentrations, while intravenous delivery achieves comparatively higher levels. Tissue distribution studies also revealed that only small amounts of unmetabolized curcumin reach organs such as the liver and kidney, further underscoring its limited absorption and rapid metabolism [239,240,241]. However, its limited systemic bioavailability prevents the achievement of therapeutically effective concentrations in vivo, which constrains its clinical translation.

**Figure 6:**
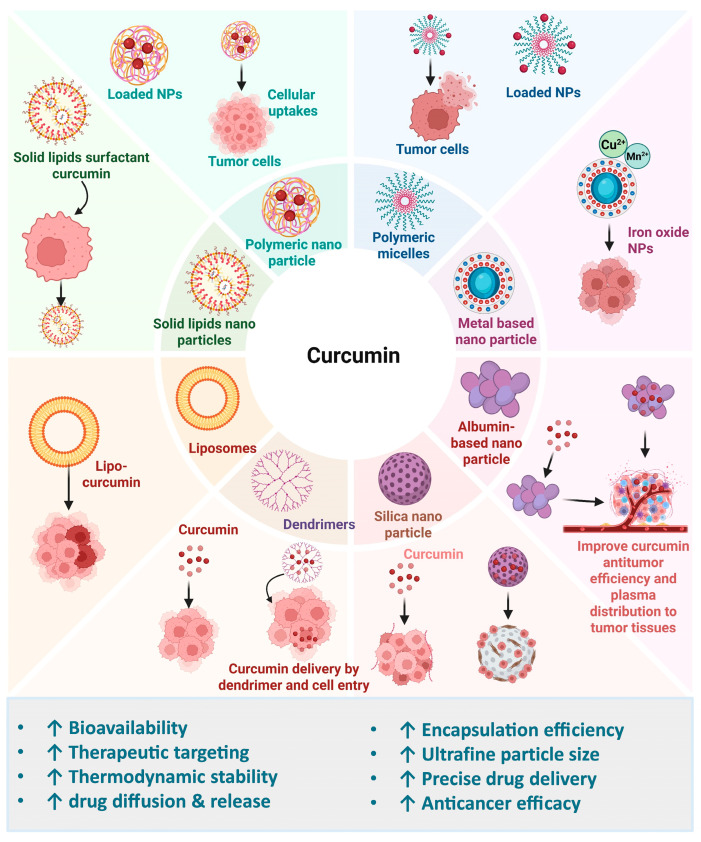
Overview of nanotechnology-based methods for delivering curcumin. Advanced systems, including liposomes, polymeric nanoparticles, solid lipid nanoparticles, albumin nanoparticles, polymeric micelles, metal-based nanoparticles, silica nanoparticles, and dendrimers, have proven effective in improving curcumin’s bioactivity [297,298,299]. The figure was prepared using Biorender.

Given these challenges, nanoformulations have been developed not as independent therapies but as strategies to overcome curcumin’s pharmacokinetic limitations, enhance efficacy, and minimize toxicity. Nanoparticle-based delivery systems are particularly effective in improving solubility, stability, and targeted delivery. The small size of nanoparticles (10–1000 nm) provides a larger surface area, boosting the interaction with solvents and improving dissolution rates, as described by the Noyes–Whitney equation. This higher surface area allows for faster dissolution of nano-sized particles. Additionally, the Ostwald–Freundlich equation explains that reducing particle size to the nanoscale can increase the drug’s saturation solubility, further enhancing its bioavailability [242,243,244,245]. Some formulations promote prolonged distribution and retention inside the body, while others prioritize the targeted delivery and internal release of drugs into cells [246,247]. Numerous studies on curcumin nanoformulations have demonstrated their potential benefits in treating various human disorders, significantly enhancing therapeutic applications (Figure 6). The limited oral bioavailability of curcumin has prompted extensive research, leading to the development of numerous innovative formulations aimed at enhancing its absorption and effectiveness [248,249,250].

**Table 2:** Summary of the several curcumin nanotechnologies and their associated drug delivery systems.

Curcumin Nanoformulation	Material Used	Target	Main Results	Ref.
Liposomes	Folic acid	Breast cancer	Induces high drug encapsulation efficiency (>73%) Exhibits higher cell toxicity Shows better cellular uptake Improves drug internalization through folate receptor overexpression	[251]
	Polyethylene glycol (PEG)	Lung cancer	Exhibits increased ability to inhibit cell growth Improves cell cycle arrest Induces marked reduction in tumor growth	[252]
	Glycyrrhetinic acid	Hepatocellular carcinoma	Exhibits higher cytotoxicity Enhanced pro-apoptotic activity Improves cellular internalization Promotes drug accumulation in tumors Suppresses drug resistance in cancer cells	[253]
	Chitosan	Hepatocellular carcinoma	Reduces cell proliferation Increases mTOR mRNA expression Exhibits significant reduction in ATG7 and Beclin-1 mRNA expression	[254]
	Glycyrrhetinic acid (GA)	Hepatocellular cancer	Enhances intracellular accumulation Promotes drug delivery into the cytosol Exhibits higher cellular toxicity Induces greater tumor growth in vivo	[255]
Polymeric nanoparticles	PEG-PLGA	Breast cancer	Exhibits high drug loading efficiency (32.22 ± 0.53%) Enhances intracellular localization Penetrates the cell membrane to improve drug delivery Induces higher cytotoxicity Reduces NF-κB protein levels Enhances tumor suppression in vivo	[256]
	Chitosan	Breast cancer	Induces significant synergistic cytotoxicity Stimulates efficient drug delivery inside the cells Increases encapsulation efficiency (87%), drug loading capacity (19.5 μg/mg) for curcumin	[257]
	Dextran	Lung cancer	Induces significant synergistic cytotoxicity, nuclear disruption	[258]
Solid lipid nanoparticles (SLNs)	Stearic acid	Lung cancer	Induces significant synergistic cytotoxicity Induces apoptosis Reduces tumor volume in vivo	[259]
	Glyceryl monostearate, stearic acid, triglycerides	Liver cancer	Enhances migration and invasion potential Reduces cell proliferation Induces apoptosis Increases uptake efficiency 3.24 times Extends the drug’s persistence time in vivo	[260]
	Surfactant	Lung cancer	Demonstrates more cytotoxicity (IC50 = 26.12 ± 1.24 µM) Induces moderate hemolytic potential towards red blood cells (RBCs) Induces higher cellular uptake from Cur-SLNs (682.08 ± 6.33 ng/µg)	[261]
	Stearic acid, glyceryl, monostearate, tristearin, Precirol ATO 5	Lung cancer	Induces significant inhibition of cell proliferation Increases tumor suppression rate (78.42%) in vivo Prevents P-glycoprotein transport Reverses multi-drug resistance	[262]
Nanomicelles	Amphiphilic block copolymers, surfactant	Breast cancer	Induces efficient cellular uptake Stimulates apoptosis, achieving impressive cell death (93%) Reverses multi-drug resistance	[263]
	CZL polymer	Liver cancer	Promotes the cellular uptake of drugs Increases apoptosis Reduces the mitochondrial membrane potential significantly Increases pro-apoptotic protein Bcl-2 Reduces antiapoptotic Bax protein expression	[264]
Silica nanoparticles	Tetraethyl orthosilicate, surfactants, 3-aminopropyltriethoxysilane	Breast cancer	Exhibits prolonged drug release Induces higher cytotoxicity Stimulates mRNA and protein expression of Bax and caspase-3/9 Reduces Bcl-2 and hTERT mRNA and protein levels	[265]
	Alginate oligosaccharide, amination	Colon cancer	Increases MSN-NH2-Cur-AOS nanoparticle (91.24 ± 1.23%) loading capacity Induces higher cytotoxicity	[266]
Protein nanoparticle (human serum albumin)	Folic acid	Esophageal cancer	Hinders cell multiplication Inhibits colony formation, Triggers cell death and cell cycle arrest Increases γ-H2AX expression	[267]
Dendrimers	Poly (amidoamine) dendrimers	Breast cancer	Demonstrates synergistic cytotoxic effects Increases apoptosis induction and cell cycle arrest Reduces Linc-RoR expression level	[268]
	Poly amidoamine dendrimer-peptide, cholesterol	Skin cancer	Induces higher encapsulation efficiency (80.2% and 76.3%) Demonstrates strong anti-skin cancer permeability	[269]
Glucan nanoparticles	β-Glucan	Hepatic cancer	Exhibits greater reduction in cell viability Stimulates ROS and NO generation Exhibits oxidative stress Induces higher encapsulation efficiency (nearly 100%)	[270]
Carbon nanotubes (CNTs)	Carbon nanotubes	Melanoma cancer	Inhibits cell proliferation Demonstrates greater cytotoxicity Induces impressive cell cycle arrest and apoptosis	[271]
	Folic acid	Ovarian cancer	Exhibits significant growth suppression Improves the drug’s cytotoxicity and cellular absorption	[272]
Metal–organic frameworks (MOFs)	Metal nodes, organic linkers	Colorectal cancer	Increases cellular uptake of therapeutics Demonstrates high encapsulation efficiency Induces massive apoptosis Induces efficient tumor regression in vivo Increases cellular uptake of the drug	[273]
	Zirconium, terephthalic acid	Breast cancer	Synergistic cytotoxic effects Upregulates Caspase 3/9 mRNA expression Downregulates CyclinD and MMP-2 mRNA expression Induce apoptosis and cell cycle arrest Suppresses migration	[274]

### 7.1. Solid Lipid-Based Curcumin Nanoparticles

In the pharmaceutical sector, solid lipid nanoparticles (SLNs) are now recognized as efficient delivery systems for a range of medicinal medicines. In the pharmaceutical industry, SLNs have become widely recognized for being effective carriers of a range of medicinal compounds. Their distinct structure improves the stability, bioavailability, and solubility of drugs, which makes them especially useful for hydrophobic substances like curcumin [275]. By enabling targeted distribution and regulated release, SLNs enhance therapeutic results. Additionally, by altering their surface characteristics, it is possible to target cells specifically, increasing therapeutic effectiveness while reducing negative effects. Colloidal carriers composed of biocompatible and biodegradable lipids, SLNs are usually between 50 and 1000 nm in size [276]. These carriers offer several advantages, including high drug loading capacity, stability, excellent biocompatibility, and improved bioavailability. SLNs serve as suitable system for sustained release and targeted delivery, especially to the reticuloendothelial system, because of their lipophilic characteristics [277,278].

Curcumin loaded into solid lipid nanoparticles has exhibited encouraging outcomes in lung cancer therapy, with the obtained results demonstrating a 12-fold reduction in tumor volume in experimental mice [259]. Additionally, curcumin encapsulated in long-circulating solid lipid nanoparticles (CU1-LSLNs) was employed to enhance its therapeutic properties and efficacy in MHCC-97H cells. The results revealed that CU1-LSLN’s capacity to induce apoptosis was greatly enhanced in MHCC-97H cells, where its absorption efficiency was 3.24 and 2.98 times more than that of free curcumin in cancer cells [260].

### 7.2. Polymeric-Based Nanoparticles

Polymeric nanoparticles (PNPs) are extremely small particles ranging from 1 to 1000 nm in size that can encapsulate drugs or be structured from a polymeric base. These nanoparticles are employed to enhance drug delivery, minimize side effects, ensure prolonged release of medications, and facilitate the administration of a wide array of therapeutic agents [279]. Natural-based polymeric nanomaterials significantly enhance biocompatibility and biodegradability, making them crucial for drug formulation. Biocompatibility is essential for minimizing toxicity to cells. These materials contribute to effective drug delivery systems while ensuring safety and compatibility with biological systems [280]. Chitosan, a biocompatible and biodegradable polysaccharide derived from crustaceans, is widely used in nanoparticle formulation [280]. The US FDA has authorized it for use as a wound dressing, and it is safe for consumption. Chitosan-based nanoformulations of curcumin enhance transdermal permeation, improve therapeutic efficacy by targeting cancer cells through various methods, increase absorption through the skin, and extend curcumin’s solubility and stability in water-based environments [281,282].

#### Polydopamine (PDA)-Based Nanoparticles

Polydopamine (PDA) is a mussel-inspired polymer with abundant catechol and quinone groups that facilitate high drug loading via π–π stacking and hydrogen bonding, simple one-pot coating, and conjugation of targeting ligands [283,284]. In addition to its excellent biocompatibility, PDA exhibits intrinsic photothermal properties, which can be harnessed for combinatorial chemo-photothermal therapy. PDA encapsulation represents a promising strategy to enhance the efficacy of curcumin-based nanomedicines. For example, PDA can form a core–shell structure around self-assembled curcumin nanoparticles through local dopamine polymerization, where morphological evolution guides optimized formulations. Beyond curcumin, PDA also serves as a generalized nanoformulation strategy for various hydrophobic drugs, providing a multifunctional platform for drug delivery, photothermal therapy, and synergistic cancer treatment. Pleiotropic applications highlight the potential of PDA as a robust nanomaterial for advancing Cur nanomedicine and broader therapeutic applications [285]. Such PDA coating improves drug stability and delivery; however, the heterogeneous nature of PDA necessitates careful evaluation of its long-term in vivo safety. Interestingly, PDA@Cur structures have also been incorporated into cotton fabrics, providing multifunctional protective effects, including antiviral, antibacterial, antioxidant, and UV-protective properties, while maintaining biocompatibility and environmental safety [286].

Polydopamine–curcumin nanoparticles (PDA-Cur NPs) further improved curcumin’s photostability and controlled release, reducing photodegradation by nearly 50% under light exposure. Compared with free curcumin, PDA-Cur NPs demonstrated enhanced antitumor efficacy by inducing apoptosis and significantly inhibiting tumor growth in MCF-7 tumor-bearing mice, with no observed systemic toxicity [287]. Mechanistically, CUR-PDA nanoparticles activated Nrf2 through inhibition of Keap1, which upregulated antioxidant and detoxification responses and attenuated liver injury in liver cancer models. CUR-PDA treatment also inhibited cancer cell migration, promoted apoptosis, and reduced pro-inflammatory cytokines (IL-1β and TNF-α) [288]. In another design, PDA@CUR@PCL/PLA injectable short fibers provided sustained and pH-responsive release of curcumin, effectively suppressing oral squamous cell carcinoma growth. The polydopamine coating also imparted strong photothermal effects, which, when combined with curcumin release, synergistically enhanced tumor cell killing and reduced tumor burden in both in vitro and in vivo models without significant toxicity [289]. Despite these advantages, batch-to-batch variability and long-term in vivo safety of PDA carriers require further evaluation.

Despite these promising results, challenges such as batch-to-batch variability and uncertainties regarding the long-term in vivo safety of PDA carriers remain to be addressed. Overall, PDA-based nanoformulations represent a pleiotropic approach that not only advances the pharmaceutical development of curcumin nanomedicines but also offers a generalized delivery strategy applicable to a wide range of hydrophobic drugs.

### 7.3. Liposome-Based Nanoparticles

Liposomes have been widely studied over the years as drug carriers and have shown significant potential for delivering curcumin in vivo [252,253,255]. Phospholipids, which form bilayer vesicles known as liposomes, are capable of encapsulating both hydrophobic and hydrophilic substances. Anticancer medications are delivered by liposomes because of their ability to change the drug components’ metabolism and excretion [290]. When liposomes are administered intravenously, the reticuloendothelial system absorbs the drug molecules that are released from the liposomes [290]. With the expanding application of liposome technology, new variants such as ligand-targeted and long-circulating liposomes have emerged. These formulations are intended to enhance the distribution of medications to different types of cancer and regulate their release into the bloodstream. Curcumin has been successfully encapsulated in liposomes using various agents, including folic acid, PEG, polymers, surfactants, and other biocompatible compounds [290].

Curcumin has been loaded into targeted chitosan-coated liposomes to accelerate the cellular breakdown of liver cancer by triggering autophagy. The results demonstrated a marked decrease in HepG2 cell proliferation following 48 h of exposure to 100 µg/mL, with a reduction of 51% ± 1.5% [254]. Additionally, an acid-sensitive component was added to an innovative liposomal formulation to improve curcumin’s selectivity for HCC cells. The results indicated that this system promoted drug release into the cytosol, and animal model experiments validated its effectivity in suppressing tumor progression [255].

### 7.4. Metalloid Nanoparticles

Sustainable chemistry has become a modern, eco-friendly alternative to traditional chemical processes. A key strategy in the realm of nanomedicines is the incorporation of different natural substances [291,292]. Metal nanoparticles (NPs) have garnered considerable attention due to their versatile properties, making them promising candidates for a variety of applications, particularly in cancer therapy. These include NPs based on materials such as iron/iron oxide, copper, gold, cerium oxide, silver, calcium, magnesium, titanium, barium, nickel, zinc, and bismuth, as documented in the scientific literature. Metal nanoparticles are increasingly integral to contemporary cancer research platforms, with growing interest in their potential. For example, a comparative analysis of folic acid-adorned curcumin-loaded iron oxide nanoparticles has shown promise as an anticancer drug delivery system. Similarly, zinc-containing metal–organic structures, synthesized by reacting Schiff base ligands with zinc ions, have demonstrated potent antitumor activity. These frameworks achieved a 26.11% curcumin encapsulation rate (79.23%) and a drug incorporation capacity of 26.11% [293]. Furthermore, a comparative analysis of metallic nanoparticles revealed that Zn-MOFs, regardless of the NO_2_ group, significantly enhance the solubility of curcumin and improve its cytotoxicity against cancer cells [294].

#### Manganese (Mn)-Based Nanoparticles

Moreover, the immunomodulatory effects of Cur have indeed been extensively explored in combination with immunostimulatory factors. Recent studies have demonstrated that Cur, when combined with immunostimulatory factors such as photothermal agents and manganese (Mn) ions, can synergistically enhance antitumor immune responses. For example, a MnO_2_-shelled nanoplatform co-loaded with Cur and doxorubicin achieved 81% inhibition of primary colorectal tumors at low doses while promoting adaptive immune responses and preventing tumor recurrence [289]. Similarly, a PDA-based nanoparticle system co-loaded with Cur and doxorubicin, combined with near-infrared photothermal therapy, inhibited primary colon tumor growth by 92% and elicited strong adaptive antitumor immunity in a rechallenge model [295]. Additionally, a pH-responsive nanoparticle system co-loading Cur, CaCO_3_, and MnO_2_ effectively reprogrammed the tumor microenvironment, inducing immunogenic cell death via ROS generation and cGAS–STING pathway activation, enhancing macrophage polarization and dendritic cell maturation, and improving αPD-1 immunotherapy outcomes [296]. These findings collectively demonstrate that combinatorial Cur-based strategies offer promising immunotherapeutic avenues to enhance the efficacy of conventional cancer chemotherapy.

### 7.5. Protein-Based Nanoparticles

In the field of drug delivery, protein-based nanoparticles, which are made from synthetic peptides and natural protein cages, have garnered a lot of interest. These nanoparticles are composed of subunits from either a single type of protein or a combination of different proteins, providing various functional groups that can be chemically modified for drug binding, imaging, or targeted delivery [300]. Sources for these protein-based nanomaterials are synthesized using techniques such as emulsion, electrospray, and de-solvation [301]. A mussel-inspired functional protein (MPKE) was employed to create curcumin-loaded nanoparticles (Cur-MPKE) for effective encapsulation and delivery. The MPKE protein, consisting of a mussel-derived module and a zwitterionic peptide, forms tightly bound nanoparticles with curcumin through hydrogen bonding and dynamic imide bonds. These Cur-MPKE nanoparticles exhibited enhanced solubility and stability in aqueous environments, along with excellent biocompatibility. Additionally, they demonstrated pH-sensitive release and increased curcumin uptake by tumor cells, amplifying both its antioxidant and antitumor properties. In vivo experiments in rats further confirmed Cur-MPKE effectively inhibited tumor expansion and cell division without inducing toxicity [302].

### 7.6. Polymeric Nanomicelle-Based Nanoparticles

Polymeric micelles have garnered significant attention as nanocarriers for therapeutic applications in cancer treatment among the wide variety of available options. Block copolymers, which are composed of both hydrophilic and hydrophobic monomer units, are used to form polymeric micelles. Hydrophobic medications can be effectively incorporated into polymeric micelles, which have a core–shell structure. The shell, made of hydrophilic polymers, provides steric protection to the micelles, preventing protein adsorption and the attachment of other adhesive cells. It is worth noting that polymeric micelles offer vast opportunities and advantages for improving the therapeutic efficacy of anticancer medications that are encapsulated.

Curcumin is a potent inhibitor of the synthesis of angiogenic growth factors, which are essential for the development of new blood vessels. Therefore, curcumin-encapsulated nanomicelles (CUR-NMs) enhance cellular uptake and cytotoxicity in cisplatin-resistant human oral cancer cells. These nanoparticles demonstrated a high entrapment efficiency (82.2%), entrapment content (147.96 µg/mL), and a mean zeta potential of −17.5τ, indicating moderate stability. Cellular uptake and cytotoxicity studies showed that CUR-NMs exhibited significantly higher cytotoxicity and uptake in both cisplatin-resistant and parental oral cancer cells and confirmed a higher percentage of apoptotic cells with CUR-NMs (31.14%).

## 8. Conclusions and Future Perspectives

Curcumin, a natural phenolic compound derived from the dietary spice turmeric, is recognized for its protective, immune-modulating, and antimicrobial effects. Notably, it also exhibits strong anticancer effects against various human cancers [303]. Curcumin’s capacity to control several signaling pathways mediates its anticancer effects. Research shows that curcumin can influence key pathways in cancer cells, including Wnt/β-catenin, PI3K/Akt, JAK/STAT, MAPK, and p53 pathways. The bioavailability of curcumin after oral consumption is limited due to its hydrophobic properties, rapid metabolism, and quick elimination by the liver. Several strategies have been developed to address this issue and improve curcumin’s systemic absorption. These include curcumin nanodrug delivery systems in liposomal forms, nanoparticles, and derivatives or structural analogs. Table 2 presents recently developed curcumin derivatives and their effects compared to the parent compound. Notably, these derivatives have shown much greater biological activity and efficacy than curcumin. Recently, nanotechnology has become crucial in improving curcumin’s potential for cancer treatment. Encapsulating curcumin into nanopreparations enhances solubility and pharmacokinetics, reduces side effects, ensures sustained release, and improves targeting.

The future research on curcumin’s anticancer activities should focus on leveraging modern scientific developments to overcome existing boundaries and unlock its full therapeutic potential, specifically accomplishing the following: (1) study its ability to modulate the tumor microenvironment, including immunity; (2) identify biomarkers to predict curcumin-based therapies; (3) develop next-generation nanocarriers (lipid-based nanoparticles, polymeric micelles, or exosomes) for the improved bioavailability, solubility, and tumor-targeting efficiency of curcumin; (4) explore co-delivery systems with other anticancer agents for a synergistic effect; (5) develop personalized curcumin formulations; (6) investigate its role in modulating epigenetic mechanisms; (7) use artificial intelligence (AI) and machine learning to predict curcumin’s interactions with molecular targets and optimize its structure for enhanced anticancer activity; (8) investigate synergy between curcumin and other plant-derived compounds for higher anticancer effects; and (9) conduct well-designed, large-scale clinical trials for various cancers and patients to uncover their anticancer potential and safety.

## Data Availability

Not applicable.

## Abbreviations

The following abbreviations are used in this manuscript:

ROS	Reactive oxygen species
TCM	Traditional Chinese Medicine
EMT	Epithelial–mesenchymal transition
MMP	Matrix metalloproteinase
GSK3β	Glycogen synthase kinase 3β
CK1α	Casein kinase 1α
FZD	Frizzled
TGF-β1	Transforming growth factor-beta 1
DAC	5-aza-2′-deoxycytidine
CRCs	Colorectal cancer cells
MDA	Malondialdehyde
5-FU	5-fluorouracil
SLNs	Solid lipid nanoparticles
Cur-MPKEs	Curcumin-loaded nanoparticles
CUR-NMs	Curcumin-encapsulated nanomicelles
CU1-LSLNs	Curcumin encapsulated in long-circulating solid lipid nanoparticles
PNPs	Polymeric nanoparticles
