# Correction: Akter et al. Revisiting Curcumin in Cancer Therapy: Recent Insights into Molecular Mechanisms, Nanoformulations, and Synergistic Combinations. *Curr. Issues Mol. Biol.* 2025, *47*, 716

**DOI:** 10.3390/cimb48050430

**Published:** 2026-04-22

**Authors:** Khadija Akter, Kainat Gul, Sohail Mumtaz

**Affiliations:** 1 Department of Plasma Bio Display, Kwangwoon University, Seoul 01897, Republic of Korea; santaafrin02@gmail.com; 2 Department of Botany, Hazara University, Mansehra 21120, Pakistan; kainat.botanist38@gmail.com; 3 Department of Chemical and Biological Engineering, Gachon University, 1342 Seongnamdaero, Sujeong-gu, Seongnam-si 13120, Republic of Korea

The authors would like to make the following correction to their published paper [1]. The change is as follows:

References [9–11,14,20,24–26,128,228,245–248,304] in the original publication are incorrectly cited and need to be replaced with the following references:

9. Aggarwal, B.B.; Harikumar, K.B. Potential therapeutic effects of curcumin, the anti-inflammatory agent, against neurodegenerative, cardiovascular, pulmonary, metabolic, autoimmune and neoplastic diseases. *Int. J. Biochem. Cell Biol.* **2009**, *41*, 40–59. https://doi.org/10.1016/j.biocel.2008.06.010.
10. Garodia, P.; Ichikawa, H.; Malani, N.; Sethi, G.; Aggarwal, B.B. From ancient medicine to modern medicine: Ayurvedic concepts of health and their role in inflammation and cancer. *J. Soc. Integr. Oncol.* **2007**, *5*, 25–37. https://doi.org/10.2310/7200.2006.029.
11. Chainani-Wu, N. Safety and Anti-Inflammatory Activity of Curcumin: A Component of Tumeric (*Curcuma longa*). *J. Altern. Complement. Med.* **2003**, *9*, 161–168. https://doi.org/10.1089/107555303321223035.
14. Miriyala, S.; Panchatcharam, M.; Rengarajulu, P. *Cardioprotective Effects of Curcumin Bt—The Molecular Targets and Therapeutic Uses of Curcumin in Health and Disease*; Aggarwal, B.B., Surh, Y.-J., Shishodia, S., Eds.; Springer: Boston, MA, USA, 2007; pp. 359–377. https://doi.org/10.1007/978-0-387-46401-5_16.
20. Gupta, S.C.; Patchva, S.; Aggarwal, B.B. Therapeutic roles of curcumin: Lessons learned from clinical trials. *AAPS J.* **2013**, *15*, 195–218. https://doi.org/10.1208/s12248-012-9432-8.
24. Zhou, X.; Afzal, S.; Wohlmuth, H.; Münch, G.; Leach, D.; Low, M.; Li, C.G. Synergistic Anti-Inflammatory Activity of Ginger and Turmeric Extracts in Inhibiting Lipopolysaccharide and Interferon-γ-Induced Proinflammatory Mediators. *Molecules* **2022**, *27*, 3877.
25. Shoba, G.; Joy, D.; Joseph, T.; Majeed, M.; Rajendran, R.; Srinivas, P.S.S.R. Influence of piperine on the pharmacokinetics of curcumin in animals and human volunteers. *Planta Med.* **1998**, *64*, 353–356. https://doi.org/10.1055/s-2006-957450.
26. Cho, H.-K.; Park, C.-G.; Lim, H.-B. Construction of a Synergy Combination Model for Turmeric (*Curcuma longa* L.) and Black Pepper (*Piper nigrum* L.) Extracts: Enhanced Anticancer Activity against A549 and NCI-H292 Human Lung Cancer Cells. *Curr. Issues Mol. Biol.* **2024**, *46*, 5551–5560.
128. Li, M.; Guo, T.; Lin, J.; Huang, X.; Ke, Q.; Wu, Y.; Fang, C.; Hu, C. Curcumin inhibits the invasion and metastasis of triple negative breast cancer via Hedgehog/Gli1 signaling pathway. *J. Ethnopharmacol.* **2022**, *283*, 114689. https://doi.org/10.1016/j.jep.2021.114689.
228. Yallapu, M.M.; Jaggi, M.; Chauhan, S.C. Curcumin nanoformulations: A future nanomedicine for cancer. *Drug Discov. Today* **2012**, *17*, 71–80. https://doi.org/10.1016/j.drudis.2011.09.009.
245. Müller, R.H.; Jacobs, C.; Kayser, O. Nanosuspensions as particulate drug formulations in therapy: Rationale for development and what we can expect for the future. *Adv. Drug Deliv. Rev.* **2001**, *47*, 3–19. https://doi.org/10.1016/S0169-409X(00)00118-6.
246. Junghanns, J.-U.A.H.; Müller, R.H. Nanocrystal technology, drug delivery and clinical applications. *Int. J. Nanomed.* **2008**, *3*, 295–310. https://doi.org/10.2147/ijn.s595.
247. Feng, T.; Wei, Y.; Lee, R.J.; Zhao, L. Liposomal curcumin and its application in cancer. *Int. J. Nanomed.* **2017**, *12*, 6027–6044. https://doi.org/10.2147/IJN.S132434.
248. Sun, J.; Bi, C.; Chan, H.M.; Sun, S.; Zhang, Q.; Zheng, Y. Curcumin-loaded solid lipid nanoparticles have prolonged in vitro antitumour activity, cellular uptake and improved in vivo bioavailability. *Colloids Surf. B Biointerfaces* **2013**, *111*, 367–375. https://doi.org/10.1016/j.colsurfb.2013.06.032.
304. Kunnumakkara, A.B.; Bordoloi, D.; Padmavathi, G.; Monisha, J.; Roy, N.K.; Prasad, S.; Aggarwal, B.B. Curcumin, the golden nutraceutical: Multitargeting for multiple chronic diseases. *Br. J. Pharmacol.* **2017**, *174*, 1325–1348. https://doi.org/10.1111/bph.13621.

Reference 237 should be removed. With this correction, the order of some references has been adjusted accordingly. The authors state that the scientific conclusions are unaffected. This correction was approved by the Academic Editor. The original publication has also been updated.
